# Administration of multipotent mesenchymal stromal cells restores liver regeneration and improves liver function in obese mice with hepatic steatosis after partial hepatectomy

**DOI:** 10.1186/s13287-016-0469-y

**Published:** 2017-01-28

**Authors:** Fernando Ezquer, Javiera Bahamonde, Ya-Lin Huang, Marcelo Ezquer

**Affiliations:** 1 Centro de Medicina Regenerativa, Facultad de Medicina, Clínica Alemana Universidad del Desarrollo, Av. Las Condes 12.438, Lo Barnechea 7710162 Santiago, Chile; 2 Departamento de Fomento de la Producción Animal, Facultad de Ciencias Veterinarias y Pecuarias, Universidad de Chile, Av. Santa Rosa 11735, La Pintana Santiago, Chile

**Keywords:** Hepatic steatosis, Liver regeneration, Multipotent mesenchymal stromal cells, Mesenchymal stem cells, Regenerative medicine

## Abstract

**Background:**

The liver has the remarkable capacity to regenerate in order to compensate for lost or damaged hepatic tissue. However, pre-existing pathological abnormalities, such as hepatic steatosis (HS), inhibits the endogenous regenerative process, becoming an obstacle for liver surgery and living donor transplantation.

Recent evidence indicates that multipotent mesenchymal stromal cells (MSCs) administration can improve hepatic function and increase the potential for liver regeneration in patients with liver damage. Since HS is the most common form of chronic hepatic illness, in this study we evaluated the role of MSCs in liver regeneration in an animal model of severe HS with impaired liver regeneration.

**Methods:**

C57BL/6 mice were fed with a regular diet (normal mice) or with a high-fat diet (obese mice) to induce HS. After 30 weeks of diet exposure, 70% hepatectomy (Hpx) was performed and normal and obese mice were divided into two groups that received 5 × 10^5^ MSCs or vehicle via the tail vein immediately after Hpx.

**Results:**

We confirmed a significant inhibition of hepatic regeneration when liver steatosis was present, while the hepatic regenerative response was promoted by infusion of MSCs. Specifically, MSC administration improved the hepatocyte proliferative response, PCNA-labeling index, DNA synthesis, liver function, and also reduced the number of apoptotic hepatocytes.

These effects may be associated to the paracrine secretion of trophic factors by MSCs and the hepatic upregulation of key cytokines and growth factors relevant for cell proliferation, which ultimately improves the survival rate of the mice.

**Conclusions:**

MSCs represent a promising therapeutic strategy to improve liver regeneration in patients with HS as well as for increasing the number of donor organs available for transplantation.

**Electronic supplementary material:**

The online version of this article (doi:10.1186/s13287-016-0469-y) contains supplementary material, which is available to authorized users.

## Background

Under normal conditions, the liver has a remarkable capacity to regenerate in order to compensate for lost or damaged hepatic tissue, a process that enables partial hepatectomy (Hpx) and living donor liver transplantation [1, 2].

Liver regeneration depends mainly on the proliferation of hepatocytes, which are quiescent under basal conditions, but maintain a unique and powerful ability to proliferate after hepatic resection or injury. Cytokines and growth factors mediate the priming of hepatocytes and promote the re-entry into the cell cycle within 24–48 hours post-Hpx. This process also triggers the induction of cell signaling that has been shown to be part of the activation of a highly orchestrated gene expression program responsible for the stepwise organization of extracellular matrix (ECM), cell proliferation, and liver growth [1, 2].

In the simplest model, each hepatocyte is expected to divide about 1.6 times after 70% Hpx, however, entry into the S phase does not necessarily mean the cell will complete its division [3]. Adult liver has many binuclear hepatocytes and their number decreases during the regenerative process [4, 5].

It must also be considered that for the successful initiation and completion of liver regeneration after Hpx, it is necessary that the remaining cells within the liver acquire sufficient substrate to support the metabolic demand for rapid proliferation [6].

Although improved perioperative care and surgical techniques allow more extended liver resections [7], pre-existing pathological abnormalities such as liver steatosis may significantly deteriorate the post-operative course after Hpx, becoming an impediment for liver surgery and living donor transplantation [3, 8–15].

For example, the mortality of Hpx patients with moderate to severe steatosis exceeds 10%, whereas the one for non-fatty liver patients is below 2% [8, 15]. Livers with more than 30% steatosis are often not used for transplantation due to an increased risk of primary non-function [11, 16, 17]. This represents a significant waste of organs, further increasing the already serious shortage of donor organs available for transplantation.

Due to the increment in prevalence of hepatic steatosis accompanied by clinical conditions like hepatocellular carcinoma, which requires surgical resection or liver transplantation, there is a persistent need for therapies that can improve post-operative liver function and regeneration.

Multipotent mesenchymal stromal cells, also referred as mesenchymal stem cells (MSCs), are a population of self-renewable and undifferentiated cells present in the bone marrow and other mesenchymal tissues of adult individuals [18].

Diverse studies have described the potential role of MSCs to promote liver regeneration after toxic injury and some clinical trials have showed that infusion of MSCs benefits patients with end-stage liver diseases [19–22]. The mechanisms responsible for these therapeutic effects are not completely understood [23–25]. It has been suggested that MSCs may engraft into the liver and transdifferentiate into hepatocytes or fuse with them [23, 24]. However, the current paradigm is that MSCs support resident progenitor cells via paracrine mechanisms [26–30].

MSC therapy has been shown to enhance hepatic regeneration following liver Hpx [31–34], however, the effect of this therapy following liver resection in an animal model of impaired hepatic regeneration, like severe steatosis, has not been investigated yet.

In this study we evaluated the role of MSCs in liver regeneration in an animal model of normal and impaired liver regeneration.

One of the oldest and most commonly used rodent models of liver regeneration is 70% Hpx, in which two thirds of the liver are surgically removed [35, 36]. One advantage of this model over others is the absence of injury to the remnant liver tissue following the regenerative stimulus (surgically induced), which thereby minimizes potential confounders for the interpretation of the functional specificity of induced signals for the regenerative response itself.

In this study we confirmed a significant inhibition of hepatic regeneration when liver steatosis was present, while the hepatic regenerative response was improved by infusion of MSCs. Specifically, MSCs restored the hepatocyte proliferative response and liver function. Furthermore, their infusion reduced apoptosis of hepatocytes, which ultimately improved the survival rate of the mice.

## Methods

### Animals and surgical procedures

Male C57BL/6 mice were housed at a constant temperature (22 ± 2 °C) and 60% relative humidity, with 12:12-hour light-dark cycle. Five-week-old mice were fed with a standard diet (normal group; 10 cal% fat, 20 cal% proteins, and 70 cal% carbohydrates) or high-fat diet (HFD) (obese group; 60 cal% fat, 20 cal% proteins, and 20 cal% carbohydrates – D12492 Research Diets Inc., New Brunswick, NJ, USA for 30 weeks. At this time, 70% Hpx was performed as previously described by Higgins and Anderson [36]. Normal and obese mice were divided into two groups that received 5 × 10^5^ MSCs (Hpx + MSCs) or vehicle (Hpx + Vh) via the tail vein immediately after Hpx. Body weight, food intake and survival rate were monitored daily, and mice were sacrificed pre-, 2 and 7 days post-Hpx. When required, animals were anesthetized with sevofluorane (Abbot Laboratories, Chicago, IL, USA). Blood samples were centrifuged, supernatant plasma was collected and finally stored at -80 °C. The livers were excised, weighed, and preserved for subsequent molecular and histological analysis.

Animal protocols were approved by the Ethics Committee of the Facultad de Medicina Clínica Alemana - Universidad del Desarrollo.

### MSC and MSC^GFP^ isolation, ex vivo expansion and characterization

Six- to eight-week-old C57BL/6 or C57BL/6-Tg ACTB-EGFP 1Osb mice were used as donors of MSCs or MSCs^GFP^ respectively. They were sacrificed by cervical dislocation and bone marrow cells were obtained by flushing femurs and tibias with sterile PBS. After centrifugation, cells were resuspended in alpha-MEM (Gibco, Waltham, MA, USA) supplemented with 10% selected fetal bovine serum (Hyclone, South Logan, UT, USA) and 80 μg/mL gentamicin (Sanderson Laboratory, Santiago, Chile), and plated at a density of 1 × 10^6^ nucleated cells per square centimeter. Non-adherent cells were removed after 72 hours by media change. When foci reached confluence, adherent cells were detached with 0.25% trypsin, 2.65 mM EDTA, centrifuged, and sub-cultured at 7000 cells per square centimeter. After two subcultures, adherent cells were characterized according to their adipogenic and osteogenic differentiation potential, as previously described [37, 38]. Briefly, to induce adipogenic differentiation, confluent adherent cells were cultured in alpha-MEM, supplemented with 1 μM dexametasone (Sigma-Aldrich, St. Louis, MO, USA), 100 μg/mL 3-isobutyl-1-meth-ylxanthine (Calbiochem, San Diego, CA, USA), 100 μM indomethacin (Sigma-Aldrich), and 0.2 UI/mL insulin (Eli Lilly, Indianapolis, IN, USA), replaced every 3 days. After 10 days of stimulation, cell differentiation into lipid-laden adipocytes was confirmed by Oil Red O staining (Sigma-Aldrich). To induce osteogenic differentiation, confluent adherent cells were cultured in alpha-MEM medium supplemented with 0.1 μM dexamethasone, 10 mM beta-glycerophosphate (Sigma-Aldrich), and 50 μg/mL ascorbate 2-phosphate (Sigma-Aldrich), replaced every 3 days. After 21 days of stimulation, cell differentiation into hydroxyapatite-producing osteoblasts was confirmed by Alizarin Red staining (Sigma-Aldrich). Although there are currently no consensus markers for murine MSCs as there exist for human MSCs [39], immunophenotyping was performed by flow cytometry analysis after immunostaining with monoclonal antibodies against lymphocyte markers B220, CD4, and CD8-Pe-Cy5 (BD Pharmingen, San Diego, CA, USA) and putative murine MSC markers SCA1-APC, CD90-PE, and CD44-PECy5 (eBioscience, San Diego, CA, USA) (Additional file 1).

### MSC or MSC^GFP^ intravenous administration

Slightly anesthetized mice received 5 × 10^5^ MSCs or MSCs^GFP^ suspended in 0.2 mL of 5% mice plasma (Hpx + MSCs) or 0.2 mL of 5% mice plasma (Hpx + Vh) via the tail vein.

### Biochemical analysis and liver histology

Serum triglycerides and cholesterol levels were determined in the Abbot Architect c8000 autoanalyzer. Blood glucose levels were measured with the glucometer system Accu-Chek Performance (Roche). Glucose tolerance test was performed as previously described [40]. To evaluate liver injury, the levels of serum aspartate aminotransferase (AST) and alanine aminotransferase (ALT) were measured via routine clinical chemistry (GOT/AST and GPT/ALT Wiener Lab, Rosario, Argentina). Serum prothrombin was measured using the ab157526-Prothrombin mouse ELISA (Abcam, Cambridge, MA, USA).

Aliquots of frozen liver were assayed for triglyceride and cholesterol measurements, as previously described [40].

For histologic analysis, serial 3-μm sections of the right lobes of the liver were stained with hematoxylin and eosin or Massons’s trichrome to evaluate hepatic steatosis and liver fibrosis respectively. Under high magnification of the × 20 objective, 20 consecutive non-overlapping fields, in each liver, were observed. A standardized score was employed to evaluate hepatic steatosis [41]: mild/uncomplicated steatosis (<30% of hepatocytes affected, without inflammatory component), moderate (30–60% of hepatocytes affected), and severe steatosis (>60% of hepatocytes affected).

### Hepatic regeneration and apoptosis assay

Three markers of liver regeneration were evaluated:

1. Restitution of the liver weight was determined as the percentage of regenerated liver mass and calculated using the following equation: liver mass regeneration (%) = 100× [C-(A-B)]/A in which A is the estimated total liver weight at the time of the partial hepatectomy, B is the weight of the excised liver, and C is the weight of the regenerated liver [3, 26].
2. Liver samples were stained for proliferating cell nuclear antigen (PCNA).
  After fixation with formalin and paraffin embedding, 4-μm-thick liver sections were evaluated by confocal microscopy as previously described [40], using anti-PCNA NB 600-1331, (Novus Biologicals, Littleton, CO, USA) and anti-rabbit IgG Fab2 Alexa Fluor 555 (Molecular Probes, Eugene, OR, USA) antibodies.
3. Liver sections were stained for 5-bromo-2-deoxy-uridine (BrdU) incorporation. A total of 100 mg/kg BrdU was injected intraperitoneally 2 hours before tissue sampling and detected using the BrdU labeling and detection kit I (Roche, Basel, Switzerland), according to the manufacturer’s instructions.

Hepatic apoptosis was assessed by the terminal deoxynucleotidyl transferase-mediated dUTB-biotin end-labeling (TUNEL) method using the DeadEnd™ Fluorometric System (Promega, Madison, WI, USA), according to the manufacturer’s instructions. For all immunofluorescence studies, the nuclei were counterstained with 4′-6′-diamino-2-phenylindole (DAPI) and fluorescence was evaluated by confocal microscopy (Fluoview FV10i, Olympus, Tokyo, Japan). Labeling indices were determined by two blinded investigators by counting PCNA-positive, BrdU-positive and TUNEL-positive nuclei per 100 hepatocytes in 30 high-power fields per liver and six livers per experimental group, using the ImageJ 1.34 software.

### Hepatocyte size and binucleation analysis

Because hepatocytes are epithelial cells, their outlines can be visualized after staining for actin [5]. The size and proportion of binucleated hepatocytes was evaluated in 4-μm-thick liver sections by confocal microscopy, as previously described, using anti-actin sc-1616, (Santa Cruz Biotechnology, Dallas, TX, USA) and anti-goat Alexa Fluor 555 A21432, (Invitrogen, Carlsbad, CA, USA) antibodies. The size and proportion of binucleated hepatocytes were quantitatively assessed in 30 random fields per animal.

### Liver gene expression analysis

Expression levels of ACC1, acetyl-CoA oxidase (ACO), basic fibroblast growth factor (bFGF), carnitine palmitoyltransferase I (CPT-1), cytochrome P450, family 2, subfamily E, polypeptide 1 (CYP2E1), cytochrome P450, family 4, subfamily a, polypeptide 10 (CYP4a10), cytochrome P450, family 4, subfamily a, polypeptide 14 (CYPa14), epidermal growth factor (EGF), FAT-CD36, hepatocyte growth factor (HGF), HMG-Coa, insulin growth factor 1 (IGF-1), interleukin (IL)-1β, IL-4, IL-6, IL-10, SRBP1a, SRBP-2, tumor necrosis factor alpha (TNF-α), uncoupling protein 2 (UCP-2) and glyceraldehyde 3-phosphate dehydrogenase (GAPDH) were assessed in liver samples by quantitative RT-qPCR, as described in Additional file 2. The mRNA levels of the target genes were normalized against the mRNA level of GAPDH and expressed as fold change versus the normal pre-hepatectomy (pre-Hpx) group.

### Quantification of systemic cytokines and growth factors

Measurements of cytokines (TNF-α, IL-6, IL-1β, IL-4 and IL-10) and growth factors (EGF, HGF, IGF-1 and bFGF) were assessed in 25 μl of plasma, using the MCYTOMAG 70 k and MAGPMAG 24 k assay kits (Luminex, Milliplex MAP, Millipore, Billerica, MA, USA), respectively, according to the manufacturer’s instructions. Plates were read on a Luminex 200 (Luminex Corp., Austin, TX, USA) and analyzed with the Milliplex Analyst software (VigeneTech Inc. Carlisle, MA, USA).

### Analysis of hepatic homing, proliferation and differentiation of transplanted MSCs^GFP^

To determine the distribution of MSCs^GFP^, liver sections were analyzed by confocal microscopy, as previously described [40], using anti-green fluorescent protein (GFP) sc-5384 (Santa Cruz Biotechnology) and anti-goat Alexa Fluor 488 A11055 (Invitrogen) antibodies. The relative percentage of donor MSC^GFP^ in the liver was semi-quantified by counting GFP(+) cells per 100 nuclei in 30 high-power fields per liver and three animals per experimental group (normal and obese), and experimental time point (2, 10 and 30 days after Hpx).

To determine if donor cells proliferate in the regenerated liver, sections were further stained with anti-PCNA NB600-1331 (Novus Biologicals, Littleton, CO, USA), anti-Ki-67 ab15589 (Abcam), and anti-rabbit IgG Fab2 Alexa Fluor 555 #4413S (Cell Signaling, Danvers, MA, USA) antibodies.

In vitro expanded MSCs and liver sections from normal and obese animals were evaluated by double-fluorescence staining to study the phenotype and differentiation of donor cells in the regenerated liver, using the primary antibodies: anti-vimentin sc-7557, anti-desmin sc-14026 (Santa Cruz Biotechnology), anti-F4/80 ab74383, anti-alpha-smooth muscle actin (α-SMA) ab5694 (Abcam) and anti-albumin NB110-16329 (Novus Biologicals, Littleton, CO, USA), with secondary antibodies: anti-rabbit IgG Fab2 Alexa Fluor 488 or 555 (Cell Signaling, Danvers, MA, USA), anti-goat Alexa Fluor 488 A11055 or 555 A21432 (Invitrogen, Carlsbad, CA, USA).

### Co-culture of Hepa 1-6 with MSCs and cell viability

Hepa 1-6 cells (ATCC CRL-1830) were cultured in Dulbecco’s modified Eagle’s medium (DMEM) supplemented with 10% selected fetal bovine serum (Hyclone) and 80 μg/mL gentamicin (Sanderson). To induce fat overloading of the cells, Hepa 1-6 at 75% confluence were exposed to different concentrations (0.5 mM, 1 mM and 2 mM) of a long-chain mixture of free fatty acids (FFAs), oleate and palmitate (2:1 ratio). Stock solutions of 50 mM oleate acid (Sigma-Aldrich) and 50 mM palmitate (Sigma-Aldrich) prepared in DMEM containing 1% bovine serum albumin were conveniently diluted in culture medium to obtain the desired concentrations.

Cell culturing was divided into four groups: (1) medium (DMEM without fetal bovine serum), (2) FFAs 0.5 mM, (3) FFAs 1 mM, and (4) FFAs 2 mM. A transwell system (0.4 μm pore size, Corning HTS Transwell-24 well, Corning, Corning, NY, USA), was used to avoid the contact of the cells with the MSCs. 2.5 × 10^5^ Hepa 1-6 cells were loaded into the lower chamber of the well, and 1 × 10^4^ MSCs were added to the upper chamber. The Hepa 1-6 cells were co-cultured with MSCs for 48 h, and viability of the Hepa 1-6 cells was evaluated. As a control group, Hepa 1-6 cells were cultured without MSCs and compared with the co-cultured group (n = 4).

Annexin V-FITC Apoptosis Detection Kit (BMS500FI, eBioscience) was used to evaluate the viability of Hepa 1-6 cells according to the manufacturer’s instructions. Briefly, cells were trypsinized and resuspended in their original culture medium to avoid the loss of apoptotic and necrotic cells.

Cells were incubated with annexin V-FITC for 20 minutes at room temperature and propidium iodide (PI) was added to detect late apoptosis and necrotic cells.

Cells were acquired in a Cyan ADP flow cytometer. Data were analyzed with Summit v4.3 software and results are presented as the percentage of living cells, negative for annexin V and PI.

### Statistical analysis

Data are presented as mean ± SEM. To analyze the statistical significance of intergroup differences, Krustal-Wallis test was used to compare mean values among all groups, and Mann-Whitney *U* test was used to compare mean values between two groups. *p* < 0.05 was considered statistically significant.

## Results

As shown in Additional file 3, mice fed with HFD (obese group) progressively increased their body weight. At Pre-Hpx, they almost doubled the body weight of mice in the normal group (46.6 ± 0.9 g vs. 26.8 ± 1.4 g). While the normal group showed a glucose tolerance test in the physiological range, mice in the obese group were glucose intolerant. Serum triglycerides and cholesterol, blood glucose and plasma insulin levels were increased in the obese group. Severe hepatic steatosis was evidenced histologically and biochemically, however, no sign of liver fibrosis was detected at this time point.

### MSC administration increases survival rate post-Hpx in obese mice, associated to improved liver regeneration

**Fig. 1:**
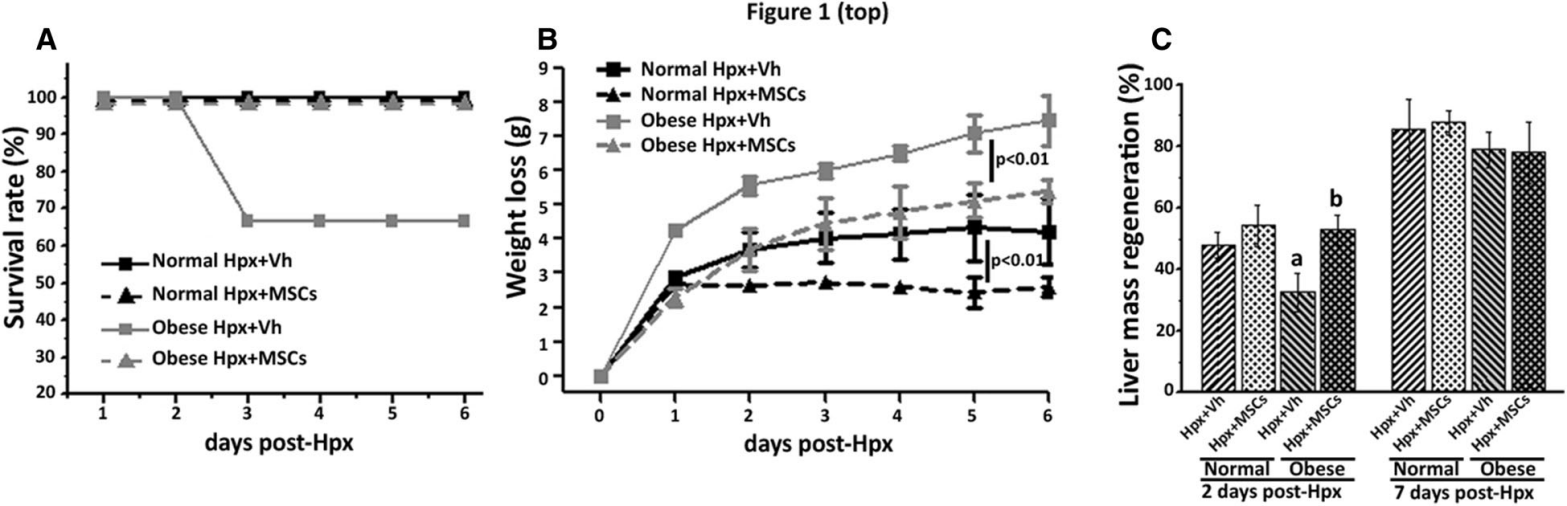
MSC administration increases survival rate and enhances liver regeneration of obese mice after 70% hepatectomy. Survival rate of mice and liver regeneration were evaluated in all experimental groups up to 7 days post-surgery. **a** Kaplan-Meier survival analysis of normal and obese mice. **b** Body weight loss post-Hpx of mice receiving vehicle or MSCs. **c** Liver regeneration represented as an increase in post-operative liver mass 2 and 7 days after surgery. All data are presented as mean ± SEM (n = 10), a *p* < 0.05 vs. normal + Vh, 2 days post-Hpx; b *p* < 0.05 vs. obese + Vh, 2 days post-Hpx

We evaluated the effect of MSC administration on animal survival after surgery. As is shown in Fig. 1a, Hpx resulted in death of 35% of obese + Vh animals (2–3 days post-Hpx), whereas all obese + MSCs mice and all mice in the normal groups (Hpx + Vh and Hpx + MSCs) survived until 7 days post-Hpx (which was the day when the mice were sacrificed).

Body weight alterations following Hpx were monitored as a marker of health fitness (Fig. 1b). Mice in the obese + Vh group showed a higher weight loss than mice in the normal + Vh group, however MSC administration significantly reduced these changes in both experimental groups.

Figure 1c shows liver regeneration rates in the four groups of mice, 2 and 7 days post-Hpx, expressed as percentage of liver mass regeneration.

In normal groups, no differences were observed in the regenerated liver mass at both time points evaluated, independent of MSC administration.

Two days post-Hpx, the rate of liver mass regeneration was lower in the obese + Vh group, compared to the normal group, however, MSC administration increased the rates up to normal group levels.

### MSC administration induces hepatocyte proliferation and reduces apoptotic rate after 70% hepatectomy

**Fig. 2:**
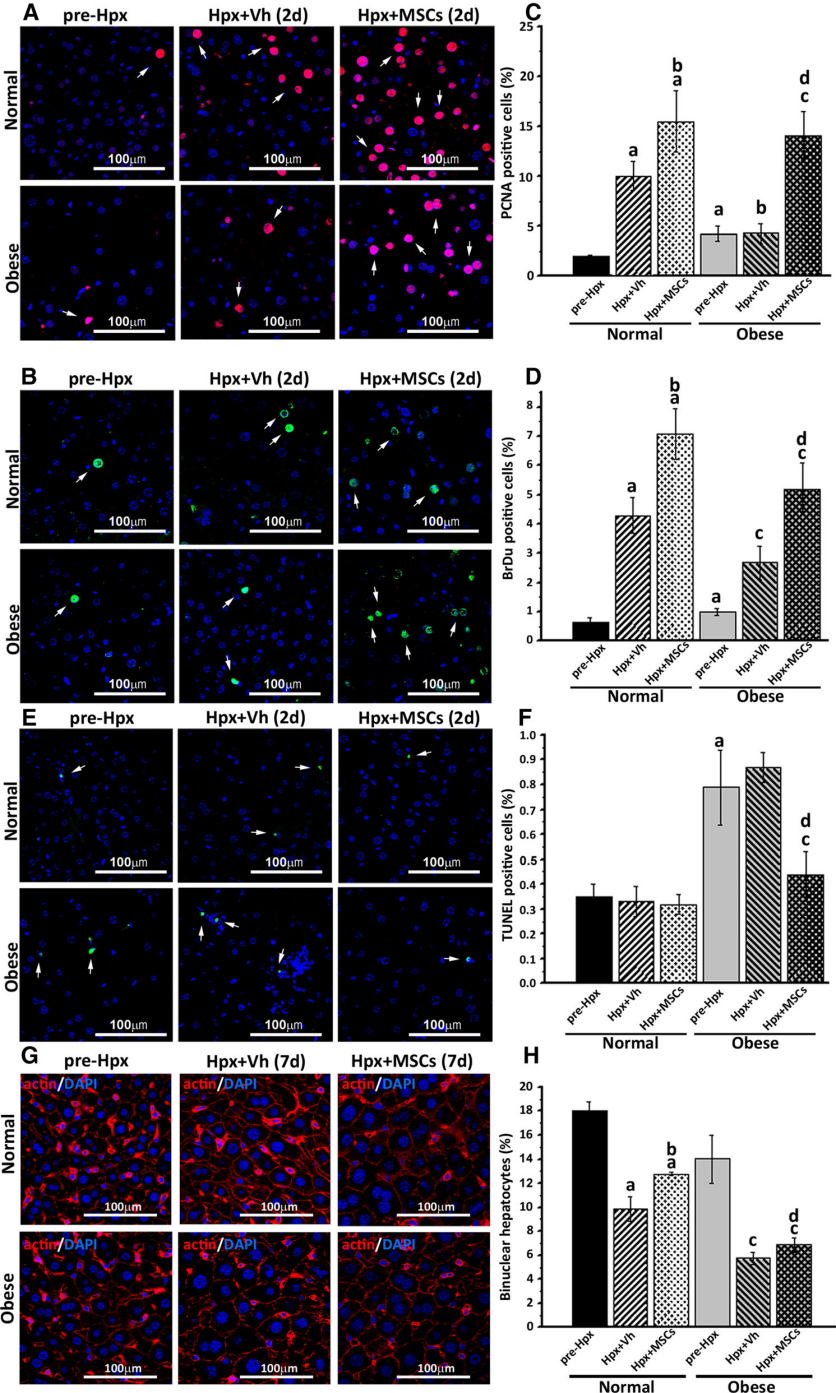
MSC administration enhances proliferation and inhibits apoptosis of liver parenchymal cells after 70% hepatectomy. Cellular proliferation and apoptosis were analyzed pre- and 2 days post liver resection in all experimental groups. The effect of MSC administration on cell proliferation was evaluated by PCNA immunoreactivity (Alexa Fluor 555 – *red*) and BrdU incorporation (Alexa Fluor 488 – *green*). Effect of MSC administration on cell apoptosis was determined by TUNEL staining (FITC – *green*). In both cases the nuclei were counterstained with DAPI (*blue*). Representative micrographs of hepatocyte proliferation determined by (**a**) PCNA labeling, or (**b**) BrdU incorporation, and apoptosis determined by (**e**) TUNEL, in liver tissue are shown (*arrows*). Quantification of (**c**) PCNA, (**d**) BrdU and (**f**) TUNEL-positive nuclei was made by digital image analysis. The proportion of mononuclear and binuclear hepatocytes at the end of the regenerative process was evaluated by immunofluorescence 7 days post-Hpx in all experimental groups. **g** Staining of outlines of hepatocytes with actin (Alexa Fluor 555 – *red*) distinguishes mononuclear from binuclear hepatocytes by confocal microscopy. **h** Quantification of binuclear hepatocytes by digital image analysis. All data are presented as mean ± SEM for 30 random fields per animal and six animals per group. a *p* < 0.05 vs. normal pre-Hpx; b *p* < 0.05 vs. normal + Vh; c *p* < 0.05 vs. obese pre-Hpx and d *p* < 0.05 vs. obese + Vh

To determine the hepatic proliferative activity, immunofluorescence staining for the proliferation marker PCNA was performed 2 days post-Hpx. As shown in Fig. 2a and c, significantly more PCNA (+) nuclei were observed in the MSC-treated versus vehicle-treated group, irrespective if the mice were normal or obese.

To further confirm the proliferation of hepatocytes, we analyzed DNA synthesis by BrdU staining. In accordance with the previous results, the percentage of BrDu (+) nuclei was higher in the MSC-treated groups (Fig. 2b, d).

Previous reports have demonstrated that 2 days after Hpx, hepatocytes are the principal proliferating cells [26, 34, 42], however, the identity of the proliferating cells was confirmed by a double immunofluorescence for albumin and BrDu incorporation (Additional file 4).

It has been reported that hepatocyte death occurs if the cells are unable to complete the mitotic cycle. TUNEL assay was used to address whether apoptosis contributes to the failure in the regeneration of fatty livers. Mice in the obese group presented an increased basal apoptotic rate pre-Hpx, which did not decrease in sections from vehicle-treated obese mice (Fig. 2e, f). Whereas, a significant reduction in TUNEL (+) nuclei was observed after MSC treatment. The determination of apoptotic cell identity is more difficult, since in the late apoptotic process detected by TUNEL, cells are irregularly shaped and have lost many of their markers (Additional file 4). However, the bibliography supports the notion that hepatocyte are the principal apoptotic cell type 2 days after Hpx [3, 12, 43]. To confirm our results, we made an in vitro co-cultured transwell study that showed that secretion from MSCs have a direct inhibitory effect on hepatic cell death (Additional file 5).

These results indicate that obese mice display impaired hepatocyte proliferation following Hpx, when compared to mice in the normal group. However, systemic administration of MSCs in this model, induces hepatocyte proliferation and reduces hepatocellular death.

To better characterize the regenerative process, the size and percentage of binuclear hepatocytes were evaluated by confocal microscopy, 7 days post-Hpx. In accordance with previous reports [5, 44], post-Hpx hepatocytes were larger in mice of the normal and obese groups, irrespective of the treatment (Additional file 6). Regarding the percentage of binuclear hepatocytes, MSC administration reduces the decrease of binuclear hepatocytes post-Hpx (Fig. 2g, h).

### MSC administration inhibits liver enzyme release and improves hepatic function after 70% hepatectomy

The levels of liver enzymes release measured in the peripheral blood provide a good estimate of ongoing liver damage. In normal and obese groups, the levels of AST and ALT were measured pre-, 2 and 7 days post-Hpx. Plasma ALT was significantly increased pre-Hpx in the obese group, indicating basal hepatocellular damage.

**Fig. 3:**
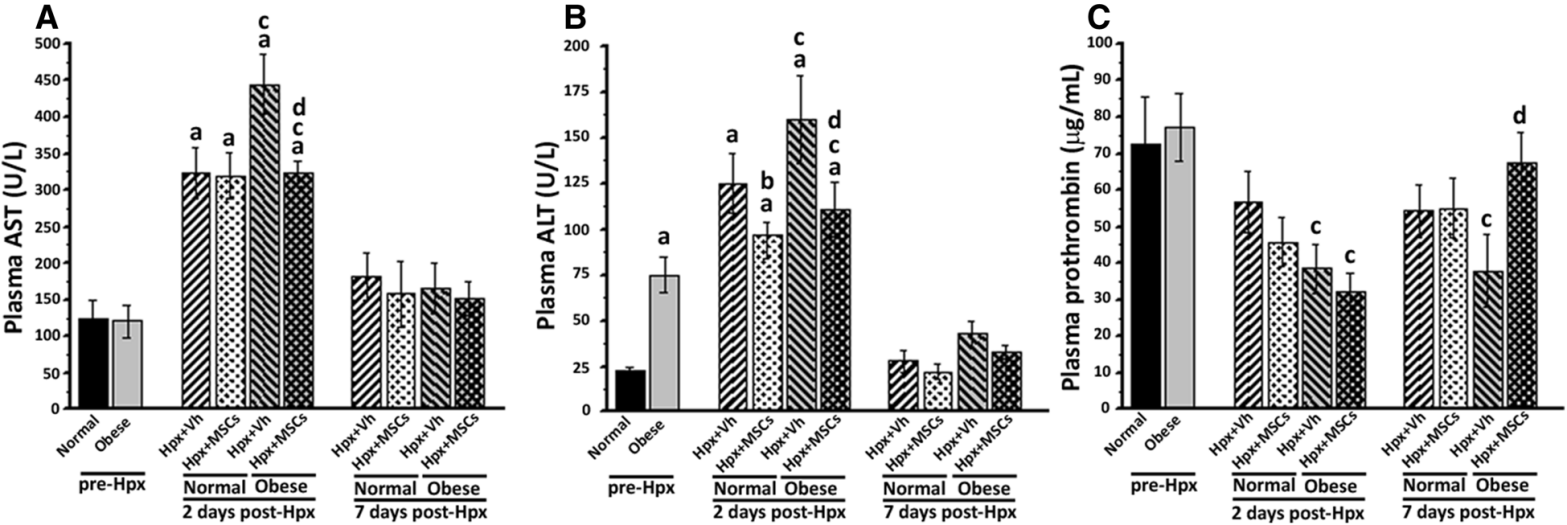
MSC administration reduces liver injury after 70% hepatectomy. Plasmatic levels of hepatocellular damage markers (**a**) aspartate aminotransferase and (**b**) alanine aminotransferase and hepatocyte function (**c**) prothrombin were determined 2 and 7 days after surgery. All data are presented as mean ± SEM (n = 8), a *p* < 0.05 vs. normal pre-Hpx; b *p* < 0.05 vs. normal + Vh; c *p* < 0.05 vs. obese pre-Hpx and d *p* < 0.05 vs. obese + Vh

We observed an expected increase in the plasmatic levels of both transaminases in mice of the normal and obese groups, 2 days post-Hpx. However, the maximum AST level was reduced significantly in the obese + MSCs versus the obese + Vh group. Accordingly, ALT plasma levels were lower in the normal + MSCs and obese + MSCs groups. Both aminotransferases returned to baseline levels in plasma 7 days post-Hpx in all experimental groups (Fig. 3a, b).

Liver function following Hpx was evaluated by measuring prothrombin plasmatic levels. Mice in the obese group showed a significant decrease in prothrombin level 2 days post-Hpx, indicative of a significant loss of hepatic function. However, MSC administration normalized prothrombin levels 7 days post-Hpx (Fig. 3c). Plasma albumin levels showed no remarkable change in all experimental groups after Hpx (data not shown).

Together, these findings suggest that MSC administration can prevent the loss of liver function and contribute at least in part to maintain or enhance early recovery after Hpx.

### Donor MSCs^GFP^ persist and proliferate, but do not differentiate into resident liver cells after 70% hepatectomy

**Fig. 4:**
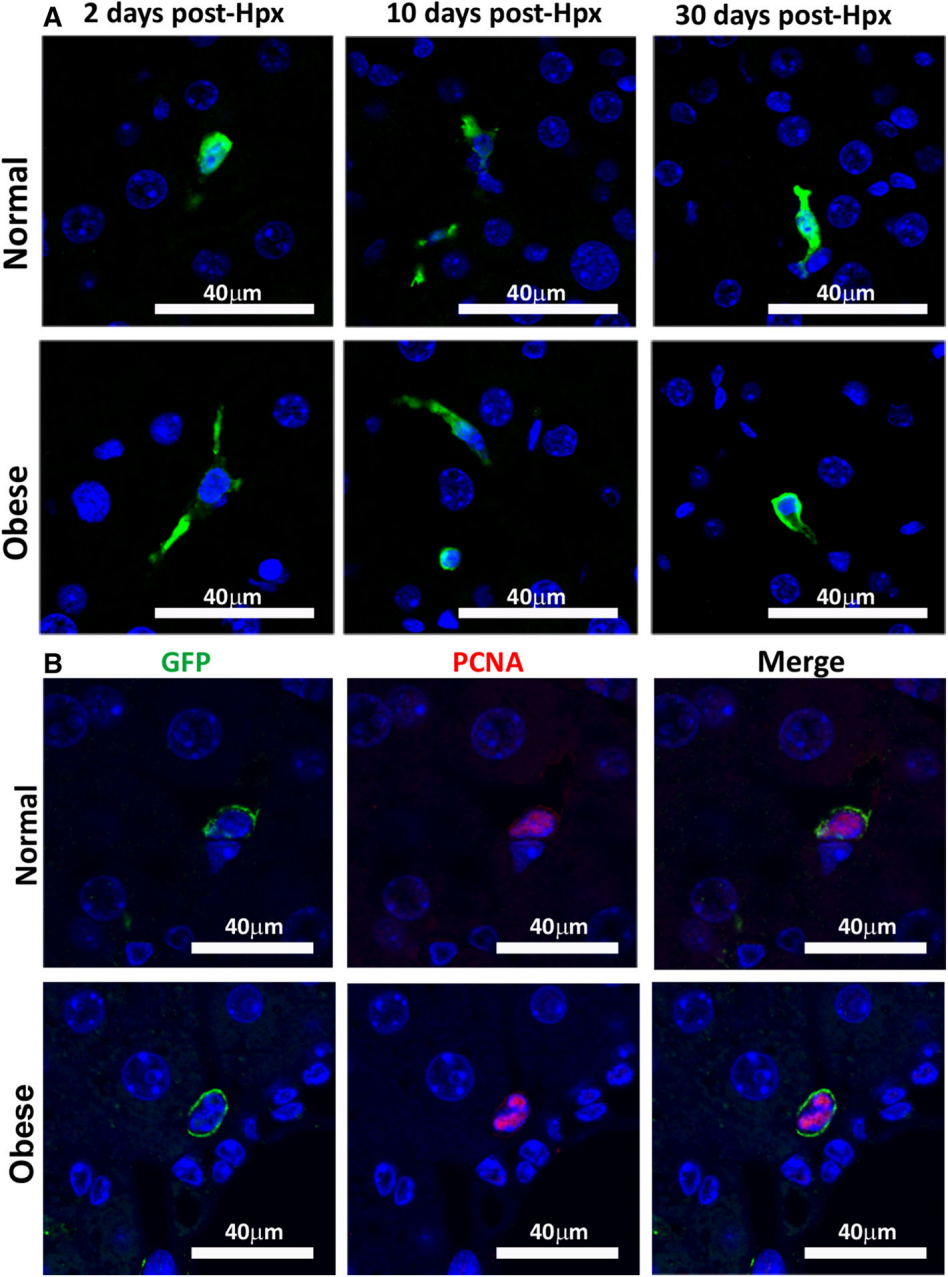
Donor MSCs persist and proliferate in the liver of 70% hepatectomized mice. Normal and obese mice received 5 × 10^5^ MSCs^GPF^ post-Hpx. Two, 10 and 30 days later, the presence of donor cells was evaluated by GFP immunoreactivity (Alexa Fluor 488 – *green*), nuclei were counterstained with DAPI (*blue*). **a** Representative micrographs of donor MSCs^GFP^ in the liver parenchyma. MSC proliferation in hepatic tissue was evaluated by PCNA immunoreactivity (Alexa Fluor 555 – *red*). **b** Representative micrographs of MSCs^GFP^ proliferation 30 days after their administration

One possibility to explain the observed therapeutic effect observed is that the administered MSCs could home to the liver and differentiate into hepatocytes or another liver resident cell type, which in turn could act as support cell to induce hepatocyte proliferation. To assess this possibility, we evaluated MSCs distribution and cellular fate after their administration using MSCs^GFP^ donor cells. Confocal microscopy analysis of liver sections from animals of the normal and obese groups, obtained after 2, 10 and 30 days post-Hpx showed a small (less than 0.3% percent) but constant number of donor single cells or cell clusters (Fig. 4a), localized in close proximity to the blood vessels, in the space of Disse (Additional file 7). Double fluorescence staining for GFP/PCNA demonstrated proliferation of donor MSCs at least 30 days after their administration (Fig. 4b).

PCNA expression has been used as a marker of cell proliferation; however, an increase in PCNA levels may also be induced by growth factors or as a result of DNA damage in the absence of cell cycling [45]. The proliferation of donor MSC was also confirmed by Ki-67 antigen expression, which is preferentially expressed during the late G1, S, G2 and M phases of the cell cycle, whereas resting, non-cycling cells lack Ki-67 expression (Additional file 8).

To evaluate whether MSCs could differentiate into liver resident cells, mature hepatocyte (albumin) or Kupffer cell (F4/80) marker were analyzed in MSCs^GFP^, however, they did not express any of these markers at any of the studied time points (Additional file 9).

### Donor MSCs change their phenotype in the recipient liver

Stellate cells (SCs) are located in the space of Disse, have features of stem/progenitor cells, and have even been considered as liver MSCs by some authors [46]. According to their phenotype, SCs can participate in both liver fibrosis and regenerative processes [47]. Since MSCs share many markers with SCs, and since in our study donor cells were found in the same hepatic niche, we evaluated by confocal microscopy the immunoreactivity of common pro-fibrogenic (α-SMA) and pro-regenerative markers (desmin and vimentin) in ex vivo expanded MSCs, before their administration, and in vivo, in liver sections 2 days post-administration.

**Fig. 5:**
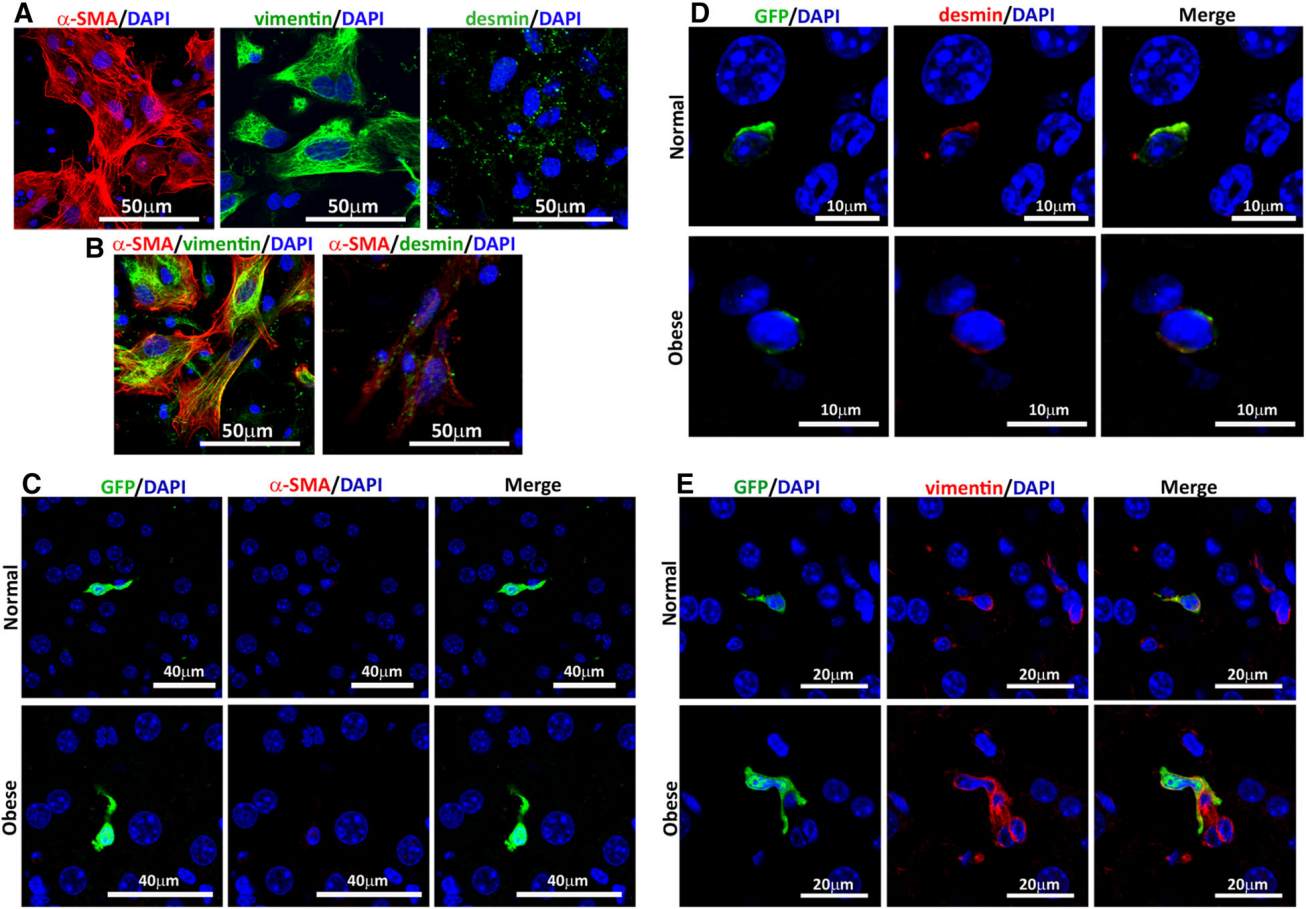
Donor MSCs show a pro-regenerative phenotype when it reaches the liver parenchyma. The in vitro expression of pro-fibrotic (α-SMA) and pro-regenerative (vimentin and desmin) markers was evaluated in donor MSCs before their administration. Representative micrographs of (**a**) individual immunoreactivity for α-SMA (Alexa Fluor 555 – *red*), vimentin (Alexa Fluor 488 – *green*) and desmin (Alexa Fluor 488 – *green*), and (**b**) co-expression of these markers. The in vivo expression of α-SMA, vimentin and desmin was evaluated by confocal microscopy in liver sections 2 days post MSCs^GFP^ administration in normal and obese hepatectomized mice. Representative micrographs of (**c**) MSCs^GFP^/α-SMA (Alexa Fluor 488 – *green*/Alexa Fluor 555 – *red*), (**d**) MSCs-^GFP^/desmin (Alexa Fluor 488 –*green*/Alexa Fluor 555 – *red*) and (**e**) MSCs-^GFP^/vimentin (Alexa Fluor 488 – *green*/Alexa Fluor 555 – *red*) colocalization. In all cases nuclei were counterstained with DAPI (*blue*)

Immunofluorescence analysis showed that MSCs co-express α-SMA, desmin, and vimentin in vitro (Fig. 5a, b), but once administered, MSCs changed their phenotype and only expressed the pro-regenerative markers desmin (Fig. 5d), and vimentin (Fig. 5e). None of the donor MSCs^GPF^ co-expressed GFP together with the pro-fibrotic marker α-SMA (Fig. 5c).

### MSC administration induces hepatic expression of key genes for hepatocyte proliferation

Diverse studies have shown that IL-6, TNF-α, and IL-1β are crucial priming factors for hepatocytes to enter the cell cycle, whereas EGF, HGF, IGF-1 and bFGF are important in the proliferative phase. In the liver these factors are produced and released primarily from hepatocytes and SCs [2, 48].

MSCs are known to produce, both in vitro and in vivo, a broad range of cytokines and trophic factors associated with tissue regeneration. To evaluate whether the therapeutic effects observed after MSC administration could be related to the generation of a pro-regenerative microenvironment, the hepatic expression of several key factors was evaluated by RT-qPCR.

**Fig. 6:**
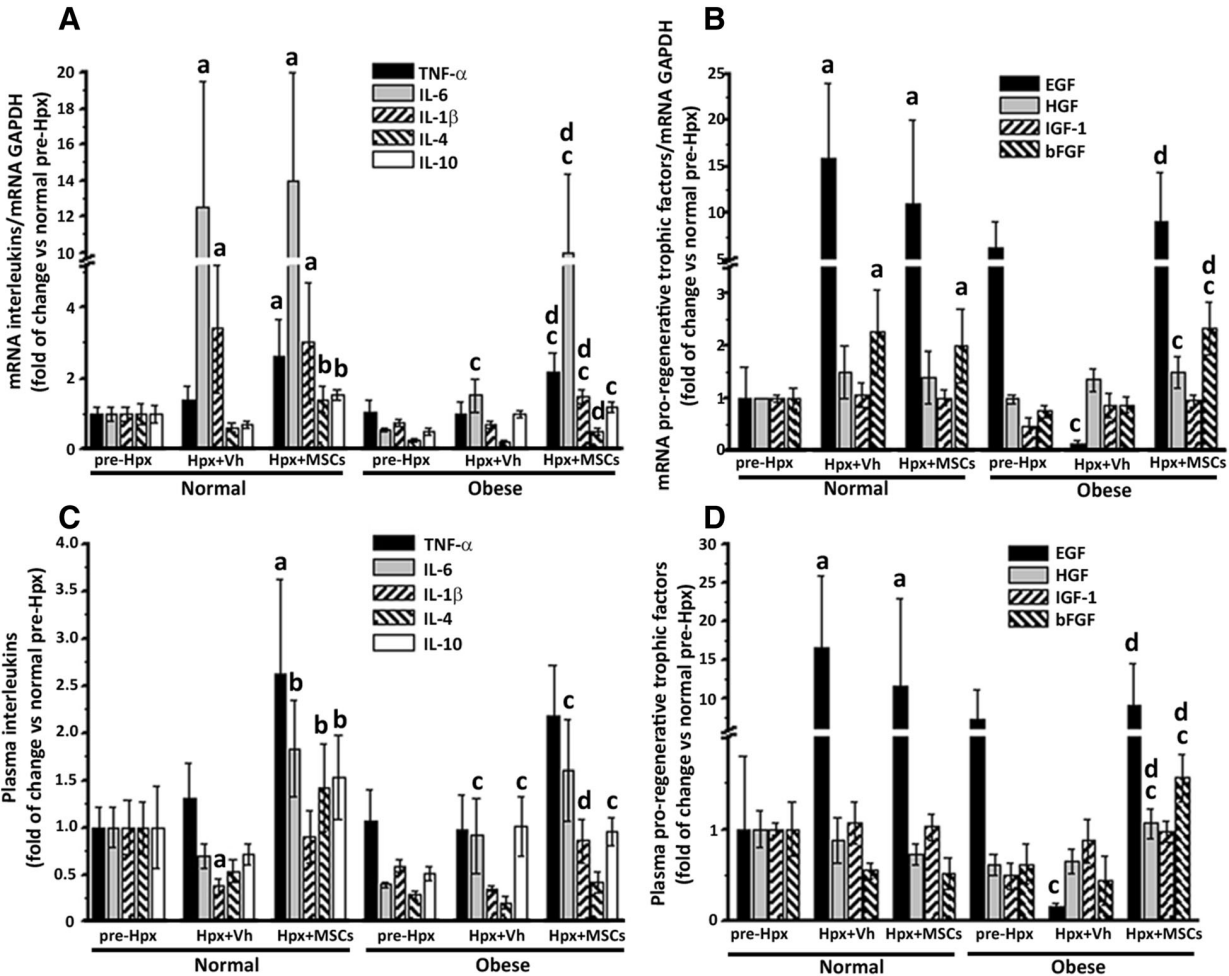
MSC administration increases local and systemic levels of key hepatic regenerative inductors, after 70% hepatectomy. Hepatic mRNA levels of (**a**) interleukins (TNF-α, IL-6, IL-1β, IL-4, and IL-10) and (**b**) pro-regenerative trophic factors (EGF, HGF, IGF-1, and bFGF) quantified by RT-qPCR, 2 days after-Hpx. Quantitative analysis of plasmatic levels of the same (**c**) interleukins and (**d**) pro-regenerative trophic factors determined by Luminex Multiplex system, 2 days after-Hpx. All data are presented as mean ± SEM (n = 8), a *p* < 0.05 vs. normal pre-Hpx; b p < 0.05 vs. normal + Vh; c *p* < 0.05 vs. obese pre-Hpx and d *p* < 0.05 vs. obese + Vh

As expected, hepatic mRNA levels of IL-6, IL-1β, EGF, and bFGF were increased 2 days after Hpx in the normal + Vh group, whereas, MSC administration additionally increased IL-4 and IL-10 expression (Fig. 6a, b). In accordance with the impaired regeneration capacity of the obese group, only IL-6 showed an increased hepatic expression 2 days post-Hpx. However, MSC administration to obese mice stimulated the liver expression of TNF-α, IL-1β, IL-4, EGF, bFGF, and potentiated the expression of IL-6 (Fig. 6a, b).

Two days post-Hpx the plasmatic levels of the above factors were also evaluated. Mice in the normal group presented increased EGF levels, while MSC administration augmented the plasmatic levels of IL-6, IL-4, IL-10, and potentiated TNF-α (Fig. 6c, d).

In the obese group an increase in IL-6 and IL-10 was observed post-Hpx in both, the vehicle- and MSC-treated groups, however, MSC administration also significantly increased the levels of IL-4, EGF, HGF, and bFGF. These results imply that the activation of liver regeneration was enhanced by MSC administration after Hpx.

### MSC administration improves hepatic β-oxidation

It has been recognized that the regenerative liver generates signals that couple fatty acid (FA) release from peripheral adipose stores to augment hepatic FA uptake, which promotes hepatic lipogenesis and leads to a rapid accumulation of intracellular triglycerides (TG) in the regenerating liver [6]. In the same line, alterations in the hepatic lipid metabolism may impair the normal liver regeneration process. For example, it has been observed that in the regenerating liver, following Hpx, the regeneration process depends on fatty acids β-oxidation responsible for energy production [6].

We evaluated the plasmatic and hepatic cholesterol and triglyceride content, 2 and 7 days post-Hpx (Additional file 10). In accordance with previous reports [49, 50], this analysis showed that the normal mice exhibited a transient increase in hepatic triglycerides 2 days post-Hpx independent of MSC administration (Additional file 10).

The obese groups presented significant steatosis pre-Hpx, however, we did not find changes in hepatic triglyceride and cholesterol levels post-Hpx (Additional file 10).

Furthermore, the administration of MSC failed to modify the expression of genes that have been associated to hepatic adipogenic changes during early liver regeneration (Additional file 10).

**Fig. 7:**
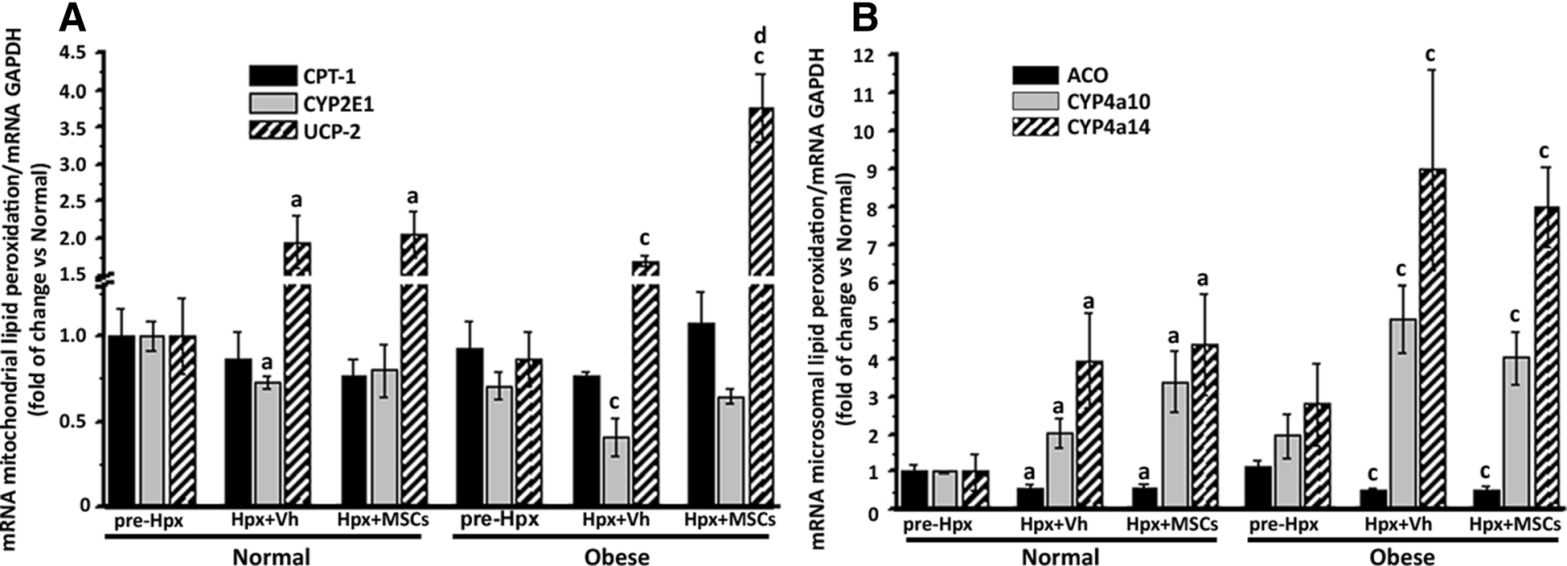
MSC administration enhances hepatic expression of UCP-2 after 70% hepatectomy in obese mice. Hepatic mRNA levels of (**a**) mitochondrial (CPT-1, CYP2E1, and UCP-2) and (**b**) microsomal (ACO, CYP4a10, CYP4a14) lipid peroxidation factors; quantified by RT-qPCR, 2 days post-Hpx. Data are presented as mean ± SEM (n = 8), a *p* < 0.05 vs. normal pre-Hpx; b *p* < 0.05 vs. normal + Vh; c *p* < 0.05 vs. obese pre-Hpx and d *p* < 0.05 vs. obese + Vh

Finally, we evaluated the hepatic expression of mitochondrial and microsomal lipid peroxidation components, 2 days post-Hpx. Administration of MSC increased the expression of UCP-2 in normal and obese groups versus vehicle-treated mice, while the hepatic expression of CYP2E1 in obese Hpx-MSCs shows the same level as pre-Hpx, the vehicle-treated group showed a significantly decrease in the CYP2E1 expression (Fig. 7). These results suggest an improvement in the β-oxidation following MSC administration.

## Discussion

The regenerative potential of the liver is essential for survival following partial resection and after acute and chronic liver injury secondary to toxins or metabolic diseases [2]. However, this potential is inhibited in hepatic steatosis, a common clinical condition resulting from a variety of etiologies such as obesity, diabetes mellitus, and alcohol intoxication [51].

There is a recognized association between chronic hepatic steatosis and impaired regeneration in experimental animal models. Diabetic KK-A [52], leptin-resistant (*db/db*) [53, 54] and leptin-deficient (ob/ob) [55, 56], high fat [9] and high fructose [57] diet-fed mice all of which exhibit hepatic steatosis, have all been reported to demonstrate impeded regeneration after hepatectomy or CCL4 administration.

In contrast to this, liver regeneration is not impaired in models of mild hepatic steatosis [58, 59], leading some investigators to postulate that the degree of steatosis is important in determining its effects on liver regeneration. Consistent with that interpretation, liver regeneration is variably affected in animals fed with a methionine choline-deficient diet, a phenotype dependent on the magnitude of steatosis [6, 12].

For testing the therapeutic effect of MSC administration, we combined a commonly used animal model of hepatic steatosis induced by chronic exposure to HFD, which is the most similar to the human clinical condition [60], and a widely validated experimental model of liver regeneration. In this animal model, in which two thirds of the liver are surgically excised and the residual liver regrows to restore most of the lost tissue and function within 5 to 7 days [35, 36, 61].

In accordance with previous reports [9], we showed that sustained feeding with a HFD resulted in impaired liver regeneration. This impairment was associated with deregulation of many of the specific signaling events known to be regulated during liver regeneration, leading to a decreased animal survival rate after Hpx.

Although a number of reports have proven that MSCs are effective in inducing hepatic regeneration [19–21, 24], and prevent non-alcoholic steatohepatitis development [40, 62, 63]. The improvement of hepatic regeneration in models of severe hepatic steatosis has not been explored.

We found that MSC administration stimulates liver regeneration in normal mice, and restores this response in obese mice after Hpx. The therapeutic effects were related to an in vivo stimulation of hepatocyte proliferation as indicated by the expression of PCNA and the incorporation of BrdU, resulting in increased growth of the remnant liver after Hpx.

Binucleation is another interesting feature of adult hepatocytes that begins in the neonatal liver [64].

It has been shown that the number of binucleated hepatocytes decreases during liver regeneration after Hpx [65]. When liver regeneration is impaired, binuclear hepatocytes seem to preferentially undergo unconventional cell divisions, in which binuclear mother cells gather their chromosomes at the center of the cells and split two nuclei to two daughter cells again [4, 5].

We evaluated the number of nuclei and confirmed that the proportion of binuclear hepatocytes decreased during liver regeneration after Hpx. However, these changes were less pronounced in normal and obese MSC-treated mice, suggesting a stimulation of hepatocyte proliferation that encourages mononuclear cells to follow the normal cell division cycle.

The significantly increased level of aminotransferases in obese untreated mice 2 days post-Hpx, along with increased apoptotic rates, indicate a vulnerability of the steatotic liver to surgical insult. Moreover, the finding that some obese + Vh mice died at this time point after Hpx suggests that these changes contributed to the poor outcome.

In spite of the increased susceptibility to liver damage due to the impaired regeneration, some obese untreated mice were able to eventually recover from Hpx, with serum aminotransferases returning to baseline levels 7 days-post Hpx, and with a liver mass similar to that of normal mice in the later phase of regeneration. In the clinical setting, however, the early post-operative phase is of greater importance, as impaired liver function immediately after Hpx makes steatotic livers more prone to develop liver failure [66, 67]. The present results, also provide evidence that steatotic livers have a significantly lower functional capacity during the entire regeneration process, since at 7 days post-Hpx, plasma prothrombin levels returned to basal levels only in obese + MSCs mice.

Both, direct differentiation of MSCs into parenchymal cells or indirect support to hepatocyte proliferation by secretion of trophic factors and cytokines, could be related to their therapeutic effects [68].

In contrast to our results, Boeykens et al. described that intraportally administered MSCs following Hpx of steatotic liver does not improve liver regeneration [69]. The differences could be associated to the hepatic steatosis model used in this work, the methionine-choline deficient (MCD) diet is not comparable to the HFD exposure, since the MCD diet model does not develop insulin resistance, and presents excessive weight and liver loss, and decreased triglycerides and cholesterol plasmatic levels, not observed in humans. Another relevant difference is the administration of MSCs 1 week after 70% Hpx, when the priming phase of hepatocytes has occurred.

Here we analyzed the migration of MSCs into the liver and their differentiation potential not only into hepatocytes, but also into macrophages and SCs, since it has been reported that these cells also participate in the regenerative process [48]. Donor-derived cells were detected in the space of Disse and proliferated in the liver of recipients. The fact that MSCs were located in this space, which has features of stem cell niches [40, 70, 71], was unexpected, since in other models of tissue injury MSCs were found near the vasculature [40, 70]. However, their failure to express albumin suggests that MSC therapeutic effects involve mechanisms other than engraftment into host tissue and differentiation into parenchymal cells.

Quiescent SCs are typically located between sinusoidal endothelial cells and hepatocytes, in the Disse space. Until recently, SCs were mainly studied regarding their fibrogenic potential in chronic diseases when they express α-SMA and acquire a myofibroblastic phenotype [47]. Conversely, their identity and function in normal liver has received little attention. In the last years, SCs have been implicated in assisting liver regeneration. Specifically, they acquire a pro-regenerative phenotype characterized by the expression of vimentin [72] and desmin [73] as well as the production of a wide array of cytokines and factors that may directly enhance the proliferation of liver progenitor cells and hepatocytes [74–77].

The evaluation of transdifferentiation of donor MSCs into SCs is complex, since in vitro cultured MSCs exhibit a marker profile similar to SCs, making discrimination between them more difficult. Moreover, recent data suggest that SCs represent liver-resident MSCs due to their potential to differentiate into adipocytes and osteocytes, and their supportive effects on extramedullary hematopoiesis [46, 78].

We confirmed that in vitro expanded MSCs express α-SMA, vimentin, and desmin. However, once administered, donor cells shut down α-SMA expression and maintain a pro-regenerative phenotype characterized by the expression of vimentin and desmin. Therefore, MSCs remaining in the Disse space could continuously deliver therapeutic molecules locally during the entire regenerative progression.

In order to initiate the liver regenerative process, the hepatocytes must first be “primed” to acquire proliferative competence by inflammatory cytokines, such as TNF-α [79] and IL-6 [3, 80], which in turn stimulate the expression of immediate early genes, enabling the cells to fully respond to growth factors like HGF, IGF-1, EGF, and bFGF [48, 81].

MSCs are known to produce and secrete, both in vitro and in vivo, a broad range of cytokines including TNF-α [82]. It has been shown that the most “upstream” event impairing hepatocyte proliferation in fatty livers is the release of TNF-α [3, 56]. Another MSC secreted cytokine, is IL-6 [29, 83], which regulates the acute-phase response in liver regeneration as well as the inhibition of hepatocyte apoptosis [84].

In accordance with previous studies [85, 86], we found another beneficial effect of MSC administration: the increased liver expression of IL-4. This response may prevent adverse effects of the pro-inflammatory cytokine TNF-α, which is needed for the initiation of liver regeneration, but also mediates cell death [87, 88].

In addition to cytokines, several growth factors, including bFGF, EGF, and HGF, are secreted by MSCs, which promote hepatocyte replication and revascularization during liver regeneration [89, 90].

Interestingly, the hepatic mRNA levels of TNF-α, IL-6, EGF, and bFGF, were significantly enhanced 2 days after Hpx in mice livers of obese MSCs-treated group compared to vehicle-treated animals, and some of these changes were additionally correlated at plasmatic level.

In the same line, we showed that non-contact co-culture in transwell caused a significantly protection of steatotic hepatic cells by MSCs. Protein-array analysis of the MSC-conditioned medium has revealed more than 150 proteins, most of which are growth factors, cytokines, and chemokines [26, 28]. In addition, extracellular vesicles, such as microvesicles and exosomes, are proposed as key mediators of information transfer between different cells for tissue repair [91]. In both cases, several of the detected molecules have known antiapoptotic and liver regeneration-stimulating effects [26, 27]. Moreover, unlike pharmaceutical treatments that deliver a single agent at a specific dose, MSCs are site-regulated and secrete bioactive factors and signals at variable concentrations in response to local microenvironmental cues [92].

We can only speculate what specific mediators present in the conditioned medium are responsible for the restoration of liver regeneration, for example factors such as HGF, bFGF, nerve growth factor, IL-Ra and IL-10, were reported to have cytoprotective or anti-inflammatory effects on hepatocytes [93]. Although, systemic proteomic analysis combined with fractionation studies of MSC-conditioned medium is necessary to identify key therapeutic components.

A number of experimental observations have suggested that alterations in systemic and hepatic lipid metabolism, including increased lipid peroxidation along with impaired mitochondrial and microsomal β-oxidation of fatty acids, and a subsequent deficit in ATP, are related to impaired liver regeneration [6, 94].

In accordance with previous studies in our laboratory [40], we found that MSC-treated groups show similar hepatic expression of CYP2E1, the major enzyme responsible for mitochondrial β-oxidation [95], than pre-Hpx groups. In contrast, the hepatic expression of CYP2E1 decreased significantly in vehicle-treated groups.

It has been suggested that the induction of CYP2E1 is an adaptive response to prevent lipid overload. It is suggestive that this result was accompanied by the increased expression of CYP4a10 and CYP4a14 in all experimental groups. Like CYP2E1, CYP4a enzymes are fatty acid hydroxylases, and are co-regulated with other genes that encode proteins involved in fatty acid β-oxidation, which in turn are key intermediates in the adaptive response to an altered hepatic lipid metabolism [95, 96].

In accordance with previous data, we confirmed increased hepatic UCP-2 mRNA levels, 2 days after Hpx [97, 98], however, MSC administration potentiated this expression. In the mitochondria, UCP-2 functions as an uncoupler when it is activated by superoxide and other metabolites of lipids and proteins [99, 100]. The proton leak via UCP-2 decreases reactive oxygen species (ROS) production and protects from oxidative stress [101–103]. In this respect, a significant delay has been described in the growth of liver remnants after Hpx in mice genetically deficient in UCP-2 [104].

Our data are not sufficient to establish a functional link between increased liver regeneration and restoration of key enzymes of β-oxidation and UCP-2, however, the sequence of these events and the fact that impaired β-oxidation and ROS have been shown to activate cell cycle inhibitory proteins [105, 106] make this relationship plausible. Further studies are needed to test this hypothesis and that issue will be taken into account in our next study.

Before translating our promising preclinical data, several practical issues should be addressed, including the best source of MSCs for transplantation. Autologous MSCs appear to be the ideal choice because they minimize infectious disease dissemination risk. However, aged MSCs display senescent features when compared with cells isolated from young donors [107, 108].

Moreover, others and we have shown that chronic diseases could modify the abundance, the phenotype, or the potentials of MSCs [109]. In particular, diet-induced obesity in mice alters the differentiation potential of MSCs resident in various tissues, including bone marrow. These effects may be regulated in part by increased levels of FFAs, but may involve other obesity-associated cytokines [110, 111]. In the same line, studies have shown that MSCs isolated from obese human donor have loss of stemness markers and increased expression of inflammatory cytokines [112, 113].

Thus, allogeneic bone marrow seems to be the ideal source of MSCs for transplantation in patients with hepatic steatosis, as in the case in the treatment of patients with other diseases, where promising results and no toxicity has been described [114].

## Conclusions

In summary, in this study we have shown that severe liver steatosis resulted in a marked inhibition of hepatic regeneration after Hpx, and that MSC administration enhanced, in normal mice, and restored, in obese mice, the hepatic regeneration process. These effects may be associated to the acquired pro-regenerative phenotype of MSCs in vivo and the upregulation of key cytokines and growth factors for cell proliferation, which ultimately improves the survival rate of the mice.

The most critical phase in patients undergoing partial liver resection or transplantation of a partial graft is during the early post-operative period. Potential new treatment strategies to enhance or accelerate liver regeneration should therefore be pointed toward this early post-operative stage. Therefore, administration of MSCs represents a promising therapeutic strategy to improve liver regeneration in patients with steatosis and to increase the number of donor organs available for transplantation.

## Additional files

Additional file 1: Characterization of bone marrow-derived MSCs isolated from C57BL/6 adult male mice. Bone marrow cells were cultured in alpha-MEM containing 10% selected fetal bovine serum into plastic dishes. Plastic adherent cells were (A) ex vivo expanded and (B) differentiated into adipogenic or (C) osteogenic lineages. (D) Cells were also immunophenotyped according to the expression of SCA-1, CD90, CD44 and no expression of B220, CD4 and CD8 antigens. Data shown are representative of cells isolated from four different animals. (PDF 232 kb)
Additional file 2: RT-PCR specific primers and characteristics of amplicons. Gene expression levels in liver samples were assessed by quantitative qRT-PCR. For this, total RNA was purified using TRIzol (Invitrogen) and quantified by absorbance at 260 nm. One microgram of total RNA was used for reverse transcription. Real-time PCR was performed in a final volume of 10 μl containing 50 ng of cDNA, PCR LightCycler-DNA Master SYBRGreen reaction mix (Roche), 3 mM MgCl2, and 0.5 μm of each primer, using a Light-Cycler thermocycler (Roche). To ensure that amplicons were from mRNA and not from genomic DNA amplification, control without reverse transcription was included. Amplicons were characterized according to their size evaluated by agarose gel electrophoresis and to their melting temperature determined in the LigthCycler thermocycler. Relative quantification was performed by the method described by Schmitten et al. [1]. (PDF 220 kb)
Additional file 3: Characterization of biochemical and histological parameters of mice exposed to HFD. After 30 weeks of exposure to regular diet (normal), or HFD (obese), several biochemical parameters were assessed, including: (A) body weight. (B) Glucose tolerance test. (C) Serum triglycerides and cholesterol, blood glucose, plasma insulin, and triglyceride content in the liver. At this time, histological analysis of liver (D) H&E and (E) Masson’s trichrome-stained sections was also performed. Data are presented as mean ± SEM, n = 15. ^*^ *p* < 0.01 vs. normal. (PDF 522 kb)
Additional file 4: Colocalization of hepatocyte and proliferation or apoptotic markers in vivo. Hepatocyte proliferation after Hpx was identified by colocalization of BrDu incorporation (FITC – *green*) and albumin (Alexa Fluor 555 – *red*). Hepatocyte apoptosis after Hpx was evaluated by colocalization of TUNEL (FITC – *green*) and albumin. Nuclei were counterstained with DAPI (*blue*). Representative micrograph of (A) proliferation and (B) apoptosis confocal microscopy 2 days after Hpx. (PDF 275 kb)
Additional file 5: MSCs improved the viability of fat overloading hepatic cells. The viability of Hepa 1-6 cells treated with 0.5, 1 or 2 mM of free fatty acid mixture (2:1 ratio of oleate and palmitate) was evaluated after 48 hours of transwell co-culture with medium (control group) or MSCs (MSCs group). All data are presented as mean ± SEM (n = 4), ^*^ *p* < 0.05 vs. control group. (PDF 154 kb)
Additional file 6: The size of the hepatocytes increases after 70% hepatectomy. The hepatocyte size at the end of the regenerative process was evaluated by immunofluorescence 7 days post-Hpx in all experimental groups. Staining of outlines of hepatocytes with actin (Alexa Fluor 555 – *red*) distinguishes the cell limits by confocal microscopy. The bar represents the hepatocyte area quantified by digital image analysis. All data are presented as mean ± SEM for 30 random fields per animal and six animals per group. a *p* < 0.05 vs. normal pre-Hpx; c *p* < 0.05 vs. obese pre-Hpx. (PDF 213 kb)
Additional file 7: Donor MSCs persist near blood vessels and space of Disse in the liver of 70% hepatectomized mice. Normal and obese mice received 5 × 10^5^ MSCs^GFP^ post-Hpx. The presence of donor cells was evaluated by GFP immunoreactivity (Alexa Fluor 488 – *green*), nuclei were counterstained with DAPI (*cyan*) and blood vessel were identified by phase contrast and marked by a *red dashed line*. Representative micrographs of donor MSCs^GFP^ 2 and 30 days after administration. (PDF 383 kb)
Additional file 8: Donor MSCs proliferate in the liver of 70% hepatectomized mice. Normal and obese mice received 5 × 105 MSCs^GPF^ post-Hpx. Two, 10 and 30 days later, the proliferation of donor cells was evaluated by colocalization of GFP (Alexa Fluor 488 – *green*) and Ki67 immunoreactivity (Alexa Fluor 555 – *red*). Nuclei were counterstained with DAPI (*blue*). Representative micrographs of donor MSC^GFP^ proliferation in the liver parenchyma 10 days after their administration. (PDF 216 kb)
Additional file 9: Donor MSCs proliferate in the liver of 70% hepatectomized mice. Normal and obese mice received 5 × 10^5^ MSCs^GPF^ post-Hpx. Two, 10 and 30 days later, the proliferation of donor cells was evaluated by colocalization of GFP (Alexa Fluor 488 – *green*) and Ki67 immunoreactivity (Alexa Fluor 555 – *red*). Nuclei were counterstained with DAPI (*blue*). Representative micrographs of donor MSC^GFP^ proliferation in the liver parenchyma 10 days after their administration. (PDF 256 kb)
Additional file 10: MSC administration does not change lipid metabolism after 70% hepatectomy. (A) Serum cholesterol level (B) serum triglycerides levels, (C) liver cholesterol content and (D) liver triglyceride content were quantified pre-, 2 and 7 days post-Hpx. Hepatic mRNA levels of key enzymes involved in (E) cholesterol (SRBP-2 and HMG-COA) and (F) triglyceride metabolism (Fat-CD36, SRBP-1a and ACC), were quantified by qRT-PCR, 2 days post-Hpx. Data are presented as mean ± SEM (n = 8), a *p* < 0.05 vs. normal pre-Hpx; b *p* < 0.05 vs. normal + Vh; c *p* < 0.05 vs. obese pre-Hpx and d *p* < 0.05 vs. obese + Vh. (PDF 376 kb)

## Abbreviations

ACO: Acetyl-CoA oxidase
ALT: Alanine aminotransferase
AST: Aspartate aminotransferase
bFGF: Basic fibroblast growth factor
BrdU: 5-bromo-2-deoxy-uridine
CPT-1: Carnitine palmitoyltransferase I
CYP2E1: Cytochrome P450, family 2, subfamily E, polypeptide 1
CYP4a10: Cytochrome P450, family 4, subfamily a, polypeptide 10
CYP4a14: Cytochrome P450, family 4, subfamily a, polypeptide 14
DAPI: 4′-6′-diamino-2-phenylindole
EGF: epidermal growth factor
FFAs: Free fatty acids
GAPDH: Glyceraldehyde 3-phosphate dehydrogenase
GFP: Green fluorescent protein
HFD: High-fat diet
HGF: Hepatocyte growth factor
Hpx: 70% hepatectomy
Hpx + MSCs: Hepatectomized mice treated with mesenchymal stem cells
Hpx + Vh: Hepatectomized mice treated with vehicle
HS: Hepatic steatosis
IGF-1: Insulin growth factor 1
IL: interleukin
MSCs: Multipotent mesenchymal stromal cells
MCD: Methionine-choline deficient
PCNA: Proliferating cell nuclear antigen
Pre-Hpx: Pre-hepatectomy
ROS: Reactive oxygen species
SCs: Stellate cells
TNF-α: Tumor necrosis factor alpha
TUNEL: Terminal deoxynucleotidyl transferase-mediated dUTB-biotin end labeling
UCP-2: Uncoupling protein 2
α-SMA: Alpha-smooth muscle actin
