# Supplementary material for: Administration of multipotent mesenchymal stromal cells restores liver regeneration and improves liver function in obese mice with hepatic steatosis after partial hepatectomy
Source: Stem Cell Res Ther. 2017 Jan 28;8:20. doi: 10.1186/s13287-016-0469-y (PMC5273822; doi:10.1186/s13287-016-0469-y)
Supplement: Additional file 1: — Characterization of bone marrow-derived MSCs isolated from C57BL/6 adult male mice. Bone marrow cells were cultured in alpha-MEM containing 10% selected fetal bovine serum into plastic dishes. Plastic adherent cells were (A) ex vivo expanded and (B) differentiated into adipogenic or (C) osteogenic lineages. (D) Cells were also immunophenotyped according to the expression of SCA-1, CD90, CD44 and no expression of B220, CD4 and CD8 antigens. Data shown are representative of cells isolated from four different animals. (PDF 232 kb) [file 13287_2016_469_MOESM1_ESM.pdf]

additional file 1 (top)

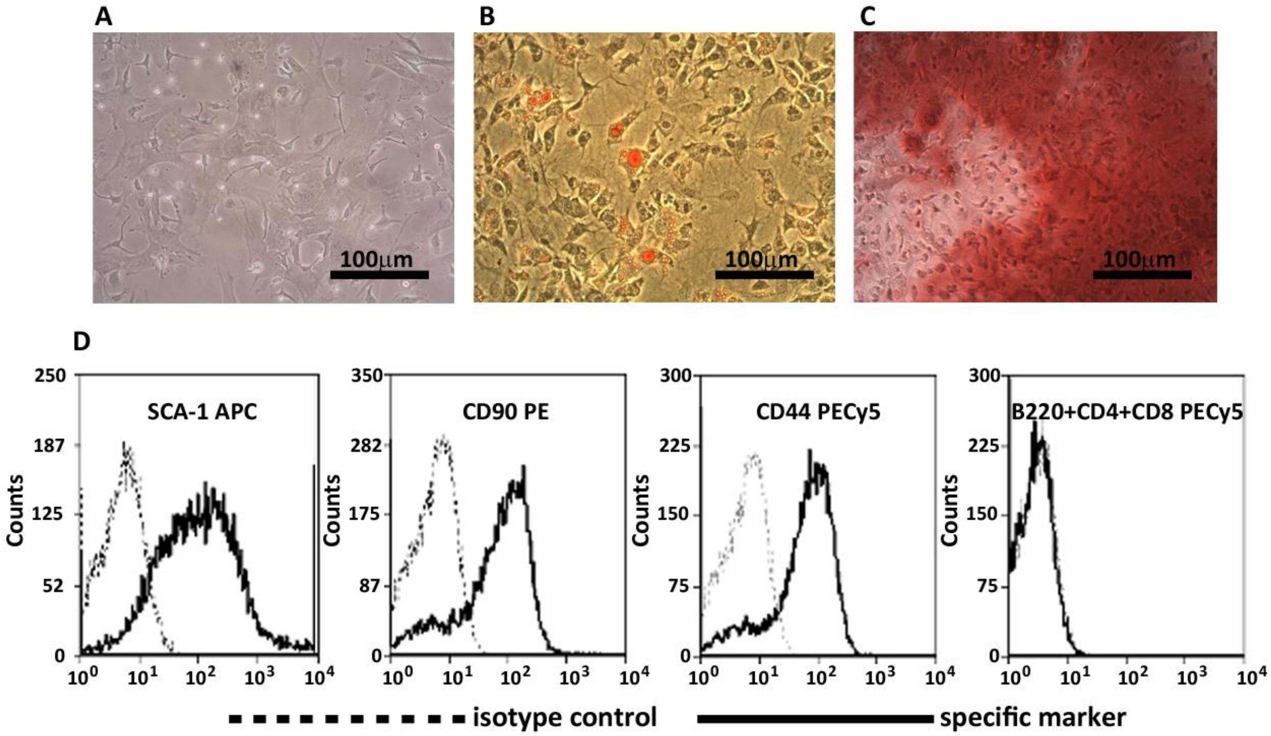

Additional file 1: *Characterization of bone-marrow-derived MSCs isolated from C57BL/6 adult male mice.*

Bone marrow cells were cultured in alpha-MEM containing 10% selected fetal bovine serum into plastic dishes. Plastic adherent cells were **(A)** *ex vivo* expanded and **(B)** differentiated into adipogenic or **(C)** osteogenic lineages. **(D)** Cells were also immunophenotyped according to the expression of SCA-1, CD90, CD44 and no-expression of B220, CD4 and CD8 antigens. Data shown are representative of cells isolated from 4 different animals.
