# Supplementary material for: Administration of multipotent mesenchymal stromal cells restores liver regeneration and improves liver function in obese mice with hepatic steatosis after partial hepatectomy
Source: Stem Cell Res Ther. 2017 Jan 28;8:20. doi: 10.1186/s13287-016-0469-y (PMC5273822; doi:10.1186/s13287-016-0469-y)
Supplement: Additional file 2: — RT-PCR specific primers and characteristics of amplicons. Gene expression levels in liver samples were assessed by quantitative qRT-PCR. For this, total RNA was purified using TRIzol (Invitrogen) and quantified by absorbance at 260 nm. One microgram of total RNA was used for reverse transcription. Real-time PCR was performed in a final volume of 10 μl containing 50 ng of cDNA, PCR LightCycler-DNA Master SYBRGreen reaction mix (Roche), 3 mM MgCl2, and 0.5 μm of each primer, using a Light-Cycler thermocycler (Roche). To ensure that amplicons were from mRNA and not from genomic DNA amplification, control without reverse transcription was included. Amplicons were characterized according to their size evaluated by agarose gel electrophoresis and to their melting temperature determined in the LigthCycler thermocycler. Relative quantification was performed by the method described by Schmitten et al. [1]. (PDF 220 kb) [file 13287_2016_469_MOESM2_ESM.pdf]

Additional file 2. RT-PCR specific primers and characteristics of amplicons.

Gene	Nº GenBank access	Sense (5'→3')	Antisense (5'→3')	Amplicon	
				size (bp)	Tm (ºC)
IL-1β	NM_008361	CAACCAACAAGTGATATTCTCCATG	GATCCACACTCTCCAGCTGCA	169	85
TNF-α	NM_013693	CATCTTCTCAAAATTCGAGTGACAA	TGGGAGTAGACAAGGTACAACCC	175	89
IL-6	NM_031168	ATCCAGTTGCCTTCTTGGGACTGA	TAAGCCTCCGACTTGTGAAGTGGT	86	134
IL-4	NM_021283	ACTTGAGAGAGATCATCGGCA	AGCTCCATGAGAACACTAGAGTT	208	86
IL-10	NM_010548	AGGGTTACTTGGGTTGCCAA	CACAGGGGAGAAATCGATGA	174	87
IGF-1	NM_001111274	TGGATGCTCTTCAGTTCGT	GTCTTGGGCATGTCAGTGT	220	84
βFGF	NM_008006	GCGACCCACACGTCAAACTA	TCCATCTTCCTTCATAGCAAGGT	101	84
EGF	NM_010113	TCTCGGATTGACCCAGAT	CCCAGACACCTTCCTCTCT	192	84
HGF	NM_010427	AAGCAATCCAGAGGTACGCTAC	TGCCATCAGGATTGCGGCAATA	224	87
FAT-CD36	NM_007643.1	ATGGGCTGTGATCGGAACTG	GTCTTCCCAATAAGCATGTCTCC	110	85
SREBP1a	NM-011480	GATGTGCGAACTGGACACAG	CATAGGGGGCGTCAAACAG	104	85
SRBP2	NM_033218.1	CCCCATGATTAAGTCCTTCAACT	GCAGCAACGGGACCATTCT	87	200
CPT-1a	NM_013495.2	CTCCGCCTGAGCCATGAAG	CACCAGTGATGATGCCATTCT	85	100
HMG-CoA	NM_008255.2	AGCTTGCCCGAATTGTATGTG	TCTGTTGTGAACCATGTGACTTC	85	104
UCP-2	NM_011671.4	ATGGTTGGTTTCAAGGCCACA	CGGTATCCAGAGGGAAAGTGAT	109	87
ACC1	NM_133360.2	ATGGGCGGAATGGTCTCTTTC	TGGGGACCTTGTCTTCATCAT	148	86
ACO	NM_015729.2	TCCAGACTTCCAACATGAGGA	CTGGGCGTAGGTGCCAATTA	286	87
CYP2E1	NM_021282.2	CGTTGCCTTGCTTGTCTGGA	AAGAAAGGAATTGGGAAAGGTCC	100	85

CYP4A10	NM_010011.3	TTCCCTGATGGACGCTCTTTA	GCAAACCTGGAAGGGTCAAAC	116	86
CYP4A14	NM_007822	TTCCCTGATGGACGCTCTTTA	GCAGCCACTGCCTTCGTAA	118	84
GADPH	XM_001474390	ACTCCACTCACGGCAAATTC	TCTCCATGGTGGTGAAGACA	171	88

RT-PCR specific primers and characteristics of amplicons.

Gene expression levels in liver samples were assessed by quantitative qRT-PCR. For this, total RNA was purified using TRIzol (Invitrogen) and quantified by absorbance at 260nm. One µg of total RNA was used for reverse transcription. Real time PCR was performed in a final volume of 10µl containing 50ng of cDNA, PCR LightCycler-DNA Master SYBRGreen reaction mix (Roche), 3mM $MgCl_2$, and 0.5µm of each primer (the above Table), using a Light-Cycler thermocycler (Roche). To ensure that amplicons were from mRNA and not from genomic DNA amplification, control without reverse transcription were included. Amplicons were characterized according to their size evaluated by agarose gel electrophoresis and to their melting temperature determined in the LigthCycler thermocycler. Relative quantification was performed by the method described by Schmitten et al (1).

Reference List

1. Schmittgen TD, Livak KJ. Analyzing real-time PCR data by the comparative C(T) method. NAT. PROTOC. 2008;3(6):1101-1108.
