# Supplementary material for: Administration of multipotent mesenchymal stromal cells restores liver regeneration and improves liver function in obese mice with hepatic steatosis after partial hepatectomy
Source: Stem Cell Res Ther. 2017 Jan 28;8:20. doi: 10.1186/s13287-016-0469-y (PMC5273822; doi:10.1186/s13287-016-0469-y)
Supplement: Additional file 3: — Characterization of biochemical and histological parameters of mice exposed to HFD. After 30 weeks of exposure to regular diet (normal), or HFD (obese), several biochemical parameters were assessed, including: (A) body weight. (B) Glucose tolerance test. (C) Serum triglycerides and cholesterol, blood glucose, plasma insulin, and triglyceride content in the liver. At this time, histological analysis of liver (D) H&E and (E) Masson’s trichrome-stained sections was also performed. Data are presented as mean ± SEM, n = 15. * p < 0.01 vs. normal. (PDF 522 kb) [file 13287_2016_469_MOESM3_ESM.pdf]

additional file 3 (top)

A

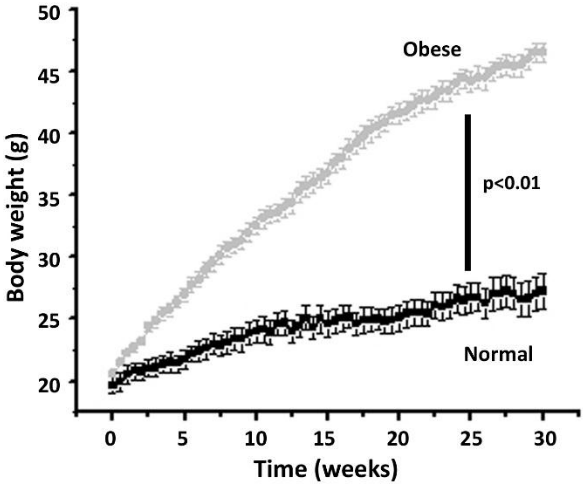

B

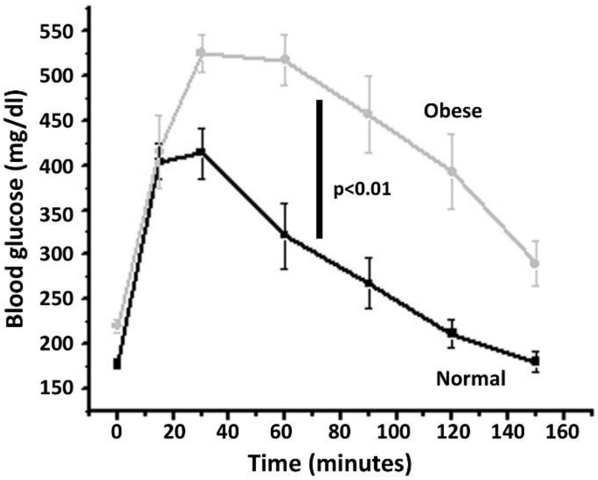

C

	Normal	Obese
Serum triglycerides (mg/dl)	74.2 ± 6.2	105 ± 8.8 *
Serum cholesterol (mg/dl)	80.2 ± 5.9	222.5 ± 13.3 *
Blood glucose (mg/dl)	140 ± 20	217 ± 10 *
Plasma insulin (µg/L)	0.6 ± 0.1	8.8 ± 2.3 *
Liver triglycerides (mg/g)	11.3 ± 2.5	76.2 ± 0.5 *
Liver total cholesterol (mg/g)	1.8 ± 0.2	3.1 ± 0.2 *

D

Normal Obese

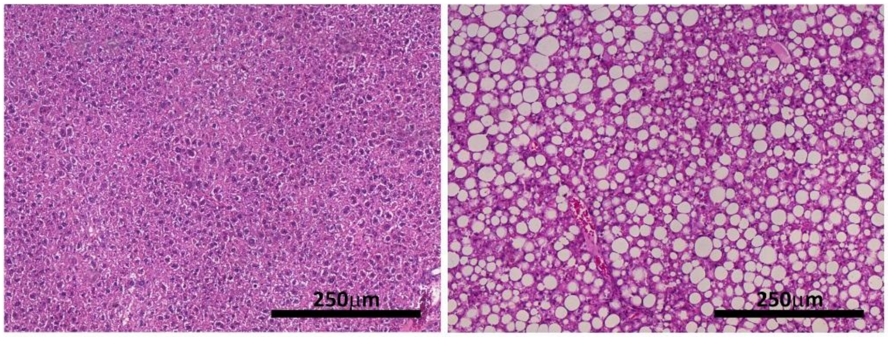

E

Normal Obese

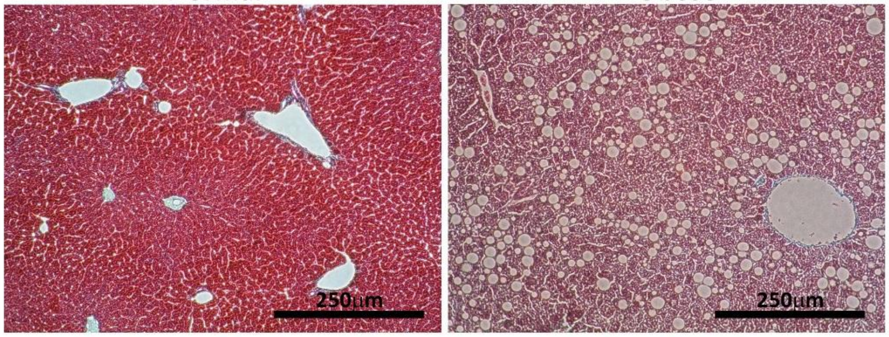

Additional file 3: *Characterization of biochemical and histological parameters of mice exposed to HFD.*

After 30 weeks of exposure to regular diet (normal), or HFD (obese), several biochemical parameters were assessed, including: **(A)** Body weight. **(B)** Glucose tolerance test. **(C)** Serum triglycerides and cholesterol, blood glucose, plasma insulin, and triglyceride content in the liver. At this time, histological analysis of liver **(D)** H&E and **(E)** Masson´s trichrome stained sections was also performed. Data are presented as mean ± S.E.M., n=15.

*p<0.01 *vs.* normal.
