# Supplementary material for: Administration of multipotent mesenchymal stromal cells restores liver regeneration and improves liver function in obese mice with hepatic steatosis after partial hepatectomy
Source: Stem Cell Res Ther. 2017 Jan 28;8:20. doi: 10.1186/s13287-016-0469-y (PMC5273822; doi:10.1186/s13287-016-0469-y)
Supplement: Additional file 4: — Colocalization of hepatocyte and proliferation or apoptotic markers in vivo. Hepatocyte proliferation after Hpx was identified by colocalization of BrDu incorporation (FITC – green) and albumin (Alexa Fluor 555 – red). Hepatocyte apoptosis after Hpx was evaluated by colocalization of TUNEL (FITC – green) and albumin. Nuclei were counterstained with DAPI (blue). Representative micrograph of (A) proliferation and (B) apoptosis confocal microscopy 2 days after Hpx. (PDF 275 kb) [file 13287_2016_469_MOESM4_ESM.pdf]

additional file 4 (top)

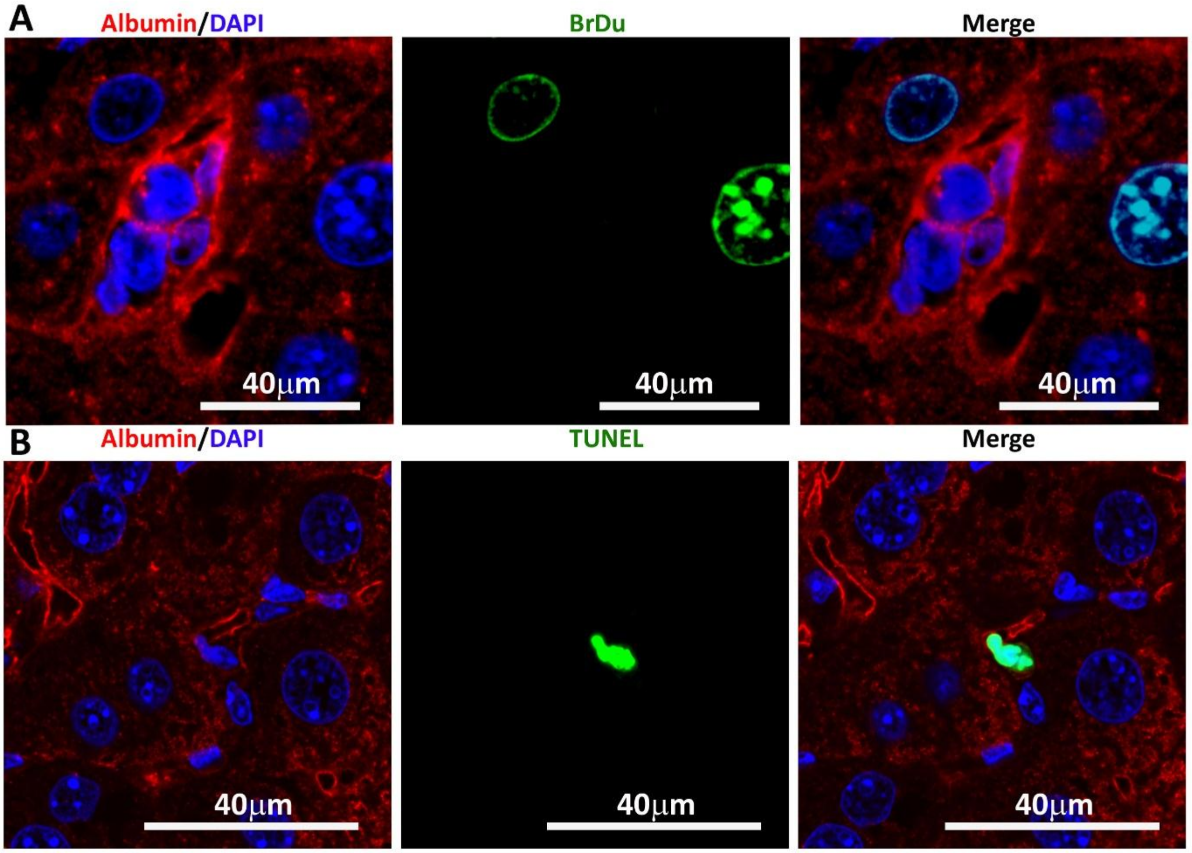

Additional file 4: *Co-localization of hepatocyte and proliferation or apoptotic markers in vivo.*

Hepatocyte proliferation after Hpx was identified by colocalization of BrDu incorporation (Fitc –green-) and albumin (Alexa Fluor 555 –red-). Hepatocyte apoptosis after Hpx was evaluated by colocalization of TUNEL (Fitc –green) and albumin. Nuclei were counterstained with DAPI (blue). Representative micrograph of **(A)** proliferation and **(B)** apoptosis confocal microscopy two days after Hpx.
