# Supplementary material for: Administration of multipotent mesenchymal stromal cells restores liver regeneration and improves liver function in obese mice with hepatic steatosis after partial hepatectomy
Source: Stem Cell Res Ther. 2017 Jan 28;8:20. doi: 10.1186/s13287-016-0469-y (PMC5273822; doi:10.1186/s13287-016-0469-y)
Supplement: Additional file 5: — MSCs improved the viability of fat overloading hepatic cells. The viability of Hepa 1-6 cells treated with 0.5, 1 or 2 mM of free fatty acid mixture (2:1 ratio of oleate and palmitate) was evaluated after 48 hours of transwell co-culture with medium (control group) or MSCs (MSCs group). All data are presented as mean ± SEM (n = 4), * p < 0.05 vs. control group. (PDF 154 kb) [file 13287_2016_469_MOESM5_ESM.pdf]

additional file 5 (top)

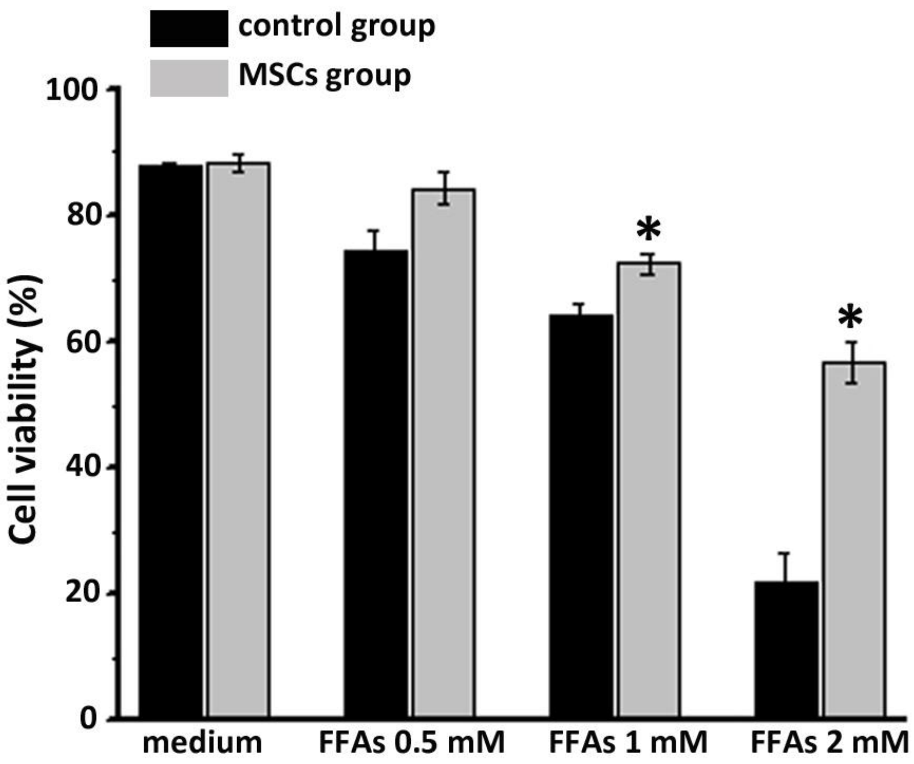

Additional file 5: *MSCs improved the viability of fat overloading hepatic cells.*

The viability of Hepa 1-6 cells treated with 0.5, 1 or 2 mM of free fatty acid mixture (2:1 ratio of oleate and palmitate) was evaluated after 48 hours of transwell co-culture with medium (control group) or MSCs (MSCs group). All data are presented as mean ± S.E.M. (n=4), * $p<0.05$ *vs.* control group.
