# Supplementary material for: Administration of multipotent mesenchymal stromal cells restores liver regeneration and improves liver function in obese mice with hepatic steatosis after partial hepatectomy
Source: Stem Cell Res Ther. 2017 Jan 28;8:20. doi: 10.1186/s13287-016-0469-y (PMC5273822; doi:10.1186/s13287-016-0469-y)
Supplement: Additional file 6: — The size of the hepatocytes increases after 70% hepatectomy. The hepatocyte size at the end of the regenerative process was evaluated by immunofluorescence 7 days post-Hpx in all experimental groups. Staining of outlines of hepatocytes with actin (Alexa Fluor 555 – red) distinguishes the cell limits by confocal microscopy. The bar represents the hepatocyte area quantified by digital image analysis. All data are presented as mean ± SEM for 30 random fields per animal and six animals per group. a p < 0.05 vs. normal pre-Hpx; c p < 0.05 vs. obese pre-Hpx. (PDF 213 kb) [file 13287_2016_469_MOESM6_ESM.pdf]

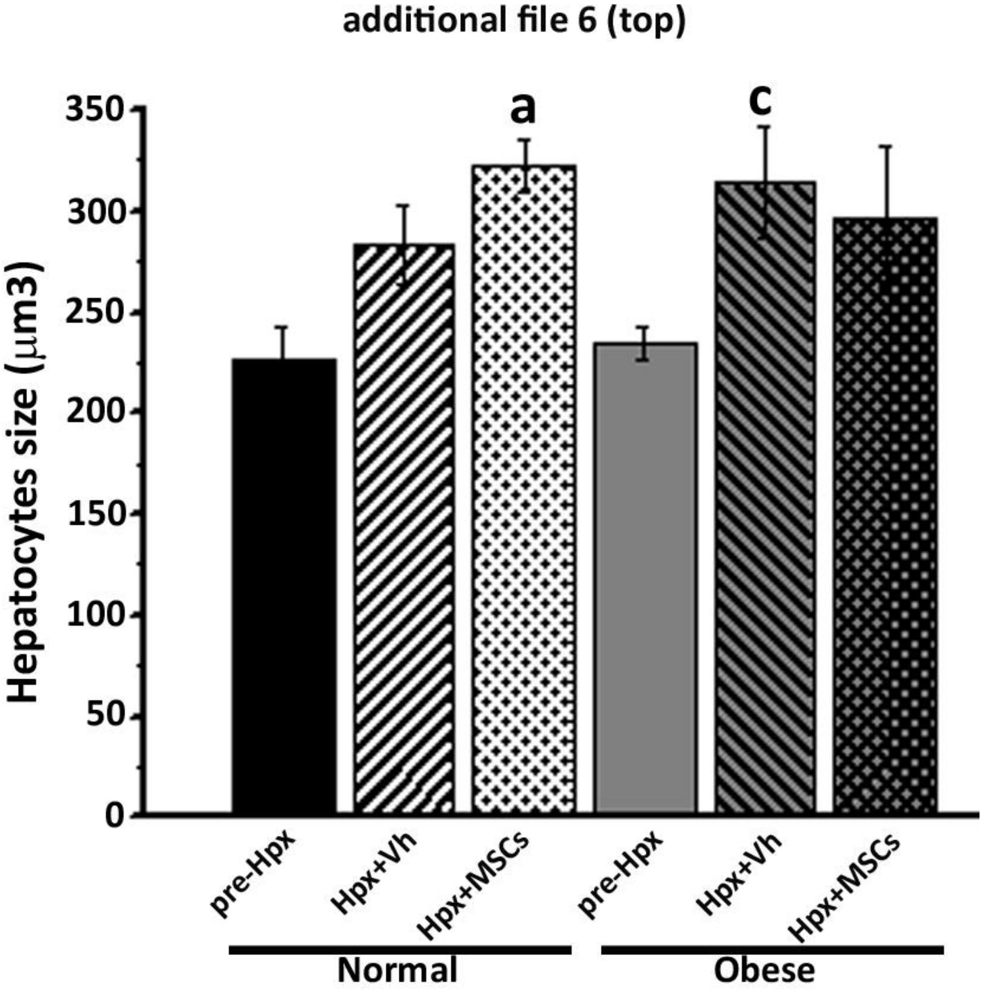

Additional file 6: *The size of the hepatocytes increases after 70% hepatectomy.*

The hepatocyte size at the end of the regenerative process was evaluated by immunofluorescence seven days post-Hpx in all experimental groups. Staining of outlines of hepatocytes with actin (Alexa Fluor 555-red-) distinguishes the cell limits by confocal microscopy. The bar represent the hepatocyte area quantified by digital image analysis. All data are presented as mean ± S.E.M. for 30 random field per animal and six animals per group. a $p<0.05$ *vs.* normal pre-Hpx; c $p<0.05$ *vs.* obese pre-Hpx.
