# Supplementary material for: Administration of multipotent mesenchymal stromal cells restores liver regeneration and improves liver function in obese mice with hepatic steatosis after partial hepatectomy
Source: Stem Cell Res Ther. 2017 Jan 28;8:20. doi: 10.1186/s13287-016-0469-y (PMC5273822; doi:10.1186/s13287-016-0469-y)
Supplement: Additional file 7: — Donor MSCs persist near blood vessels and space of Disse in the liver of 70% hepatectomized mice. Normal and obese mice received 5 × 10^5^ MSCsGFP post-Hpx. The presence of donor cells was evaluated by GFP immunoreactivity (Alexa Fluor 488 – green), nuclei were counterstained with DAPI (cyan) and blood vessel were identified by phase contrast and marked by a red dashed line. Representative micrographs of donor MSCsGFP 2 and 30 days after administration. (PDF 383 kb) [file 13287_2016_469_MOESM7_ESM.pdf]

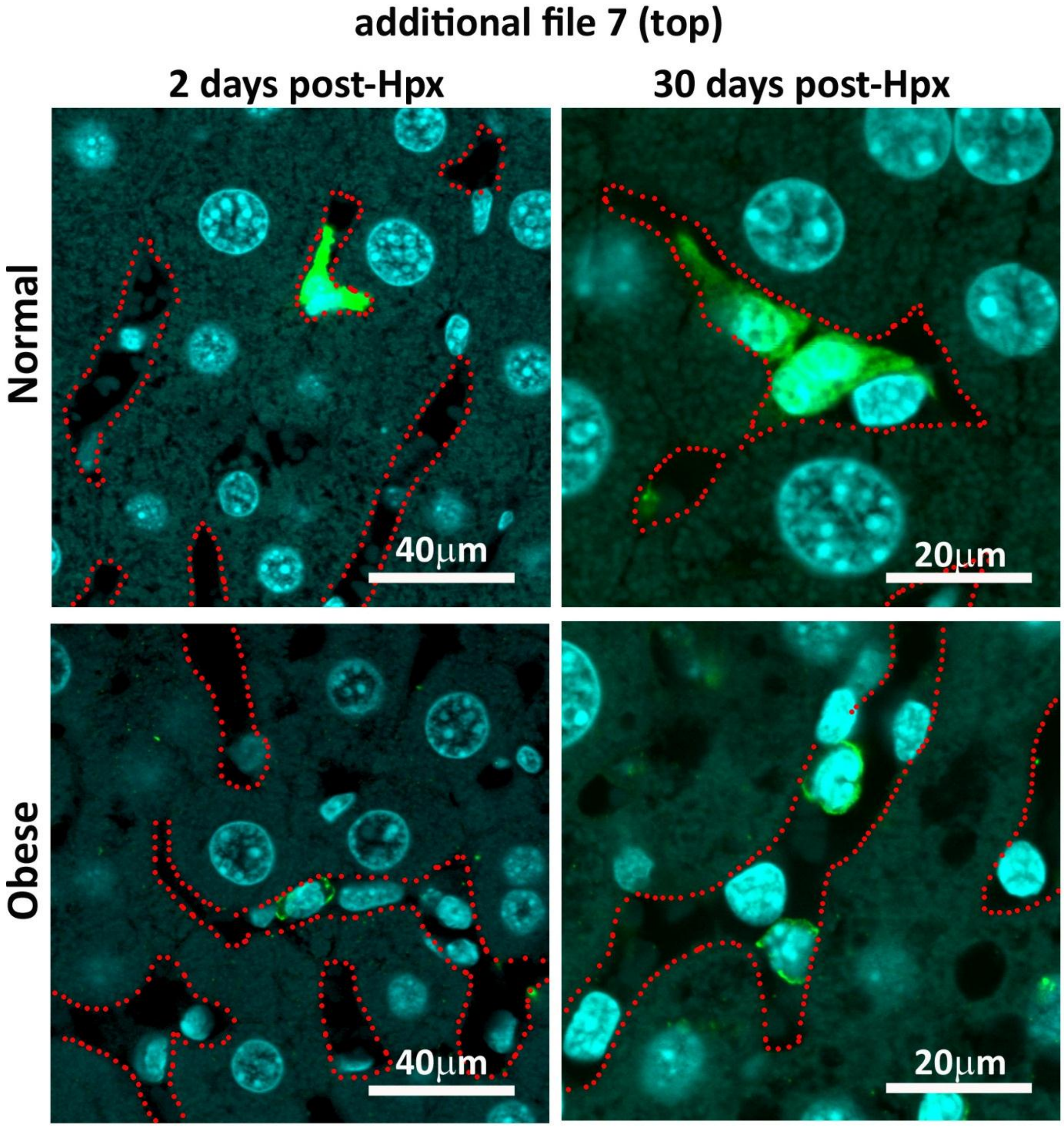

Additional file 7: *Donor MSCs persist near blood vessels and space of Disse in the liver of 70% hepatectomized mice.*

Normal and obese mice received 5 x 10^5 $MSCs^{GFP}$ post-Hpx. The presence of donor cells was evaluated by GFP immunoreactivity (Alexa Fluor 488 –green-), nuclei were counterstained with DAPI (cyan) and blood vessel were identified by phase contrast and

marked by a red dash line. Representative micrographs of donor $MSCs^{GFP}$ two and 30 days after administration.
