# Supplementary material for: Administration of multipotent mesenchymal stromal cells restores liver regeneration and improves liver function in obese mice with hepatic steatosis after partial hepatectomy
Source: Stem Cell Res Ther. 2017 Jan 28;8:20. doi: 10.1186/s13287-016-0469-y (PMC5273822; doi:10.1186/s13287-016-0469-y)
Supplement: Additional file 8: — Donor MSCs proliferate in the liver of 70% hepatectomized mice. Normal and obese mice received 5 × 10^5^ MSCsGPF post-Hpx. Two, 10 and 30 days later, the proliferation of donor cells was evaluated by colocalization of GFP (Alexa Fluor 488 – green) and Ki67 immunoreactivity (Alexa Fluor 555 – red). Nuclei were counterstained with DAPI (blue). Representative micrographs of donor MSCGFP proliferation in the liver parenchyma 10 days after their administration. (PDF 216 kb) [file 13287_2016_469_MOESM8_ESM.pdf]

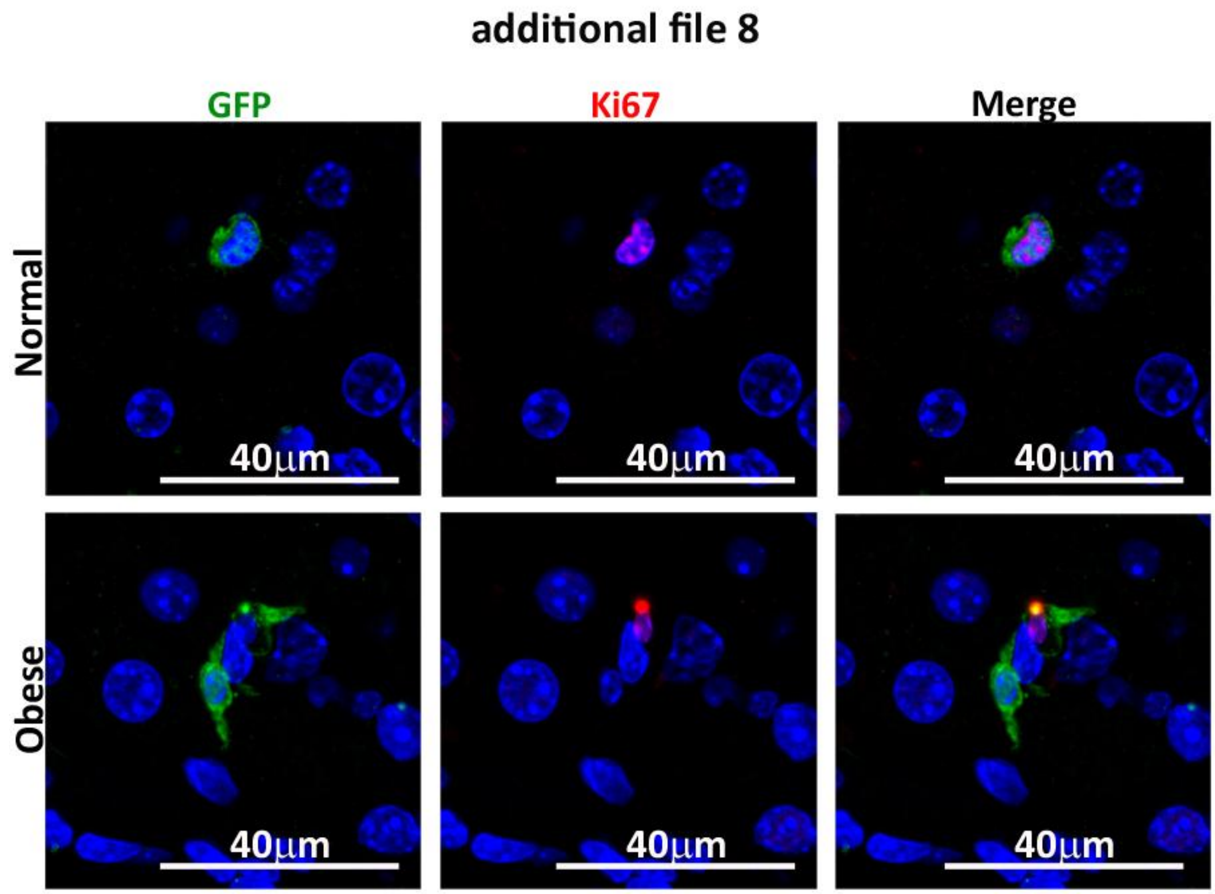

Additional file 8: *Donor MSCs proliferate in the liver of 70% hepatectomized mice.*

Normal and obese mice received 5 x 105 MSCsGPF post-Hpx. Two, ten and 30 days later, the proliferation of donor cells was evaluated by colocalization of GFP (Alexa Fluor 488 –green-) and Ki67 immunoreactivity (Alexa Fluor 555 –red-). Nuclei were counterstained with DAPI (blue). Representative micrographs of donor MSCGFP proliferation in the liver parenchyma 10 days after their administration.
