# Supplementary material for: Administration of multipotent mesenchymal stromal cells restores liver regeneration and improves liver function in obese mice with hepatic steatosis after partial hepatectomy
Source: Stem Cell Res Ther. 2017 Jan 28;8:20. doi: 10.1186/s13287-016-0469-y (PMC5273822; doi:10.1186/s13287-016-0469-y)
Supplement: Additional file 10: — MSC administration does not change lipid metabolism after 70% hepatectomy. (A) Serum cholesterol level (B) serum triglycerides levels, (C) liver cholesterol content and (D) liver triglyceride content were quantified pre-, 2 and 7 days post-Hpx. Hepatic mRNA levels of key enzymes involved in (E) cholesterol (SRBP-2 and HMG-COA) and (F) triglyceride metabolism (Fat-CD36, SRBP-1a and ACC), were quantified by qRT-PCR, 2 days post-Hpx. Data are presented as mean ± SEM (n = 8), a p < 0.05 vs. normal pre-Hpx; b p < 0.05 vs. normal + Vh; c p < 0.05 vs. obese pre-Hpx and d p < 0.05 vs. obese + Vh. (PDF 376 kb) [file 13287_2016_469_MOESM10_ESM.pdf]

additional file 10 (top)

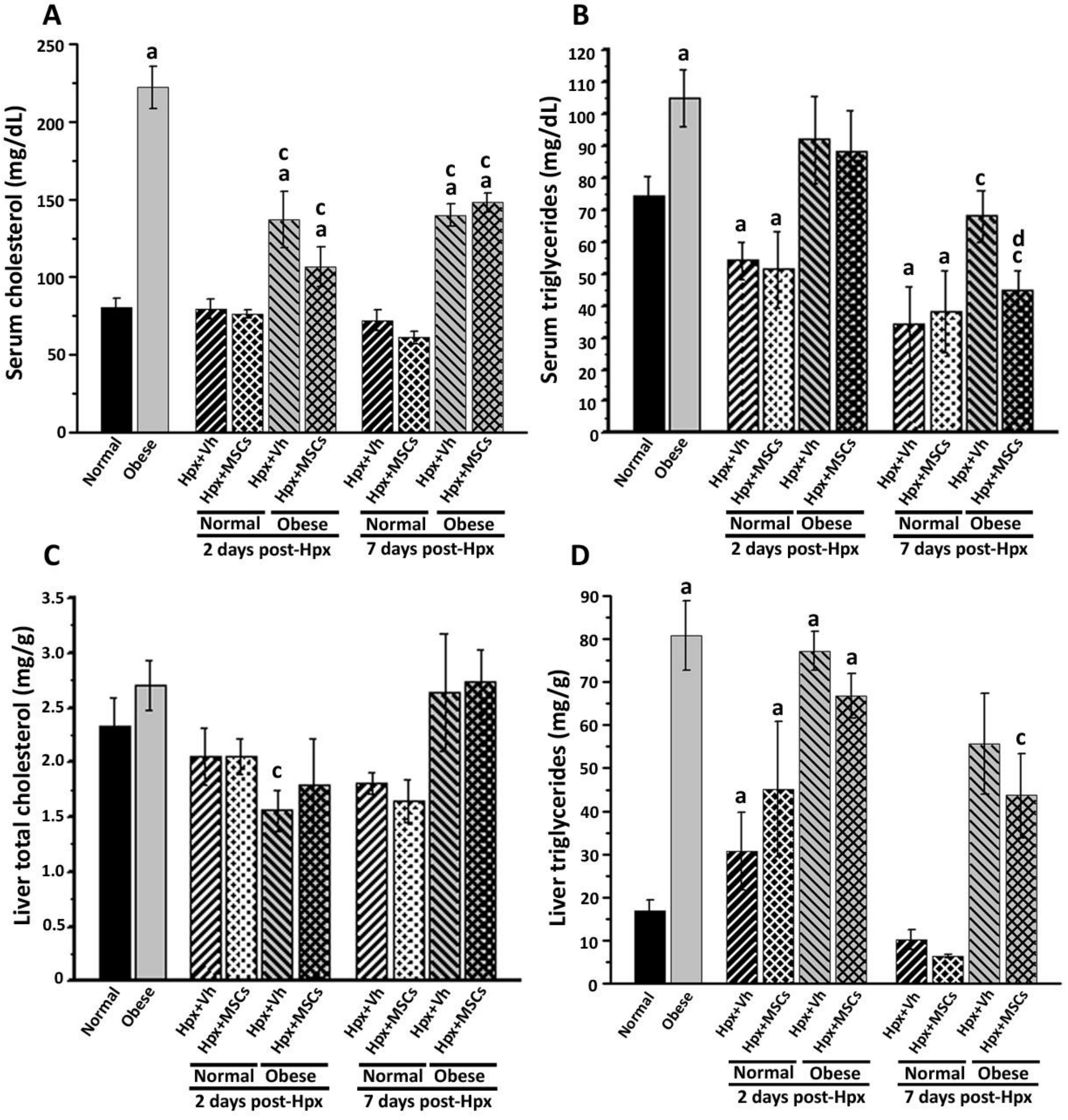

Additional file 10: *MSC administration does not change lipid metabolism after 70% hepatectomy.*

(A) Serum cholesterol level **(B)** serum triglycerides levels, **(C)** liver cholesterol content and **(D)** liver triglyceride content were quantified pre-, two and seven days post-Hpx. Hepatic mRNA levels of key enzymes involved in **(E)** cholesterol (SRBP-2 and HMG-COA) and **(F)** triglyceride metabolism (Fat-CD36, SRBP-1a and ACC), were quantified by qRT-PCR, two

days post-Hpx. Data are presented as mean ± S.E.M. (n=8), a $p<0.05$ *vs.* normal pre-Hpx; b $p<0.05$ *vs.* normal+Vh; c $p<0.05$ *vs.* obese pre-Hpx and d $p<0.05$ *vs*. obese+Vh.
